# Morphological and Taxonomic Properties of the Newly Isolated *Cotonvirus japonicus*, a New Lineage of the Subfamily *Megavirinae*

**DOI:** 10.1128/JVI.00919-21

**Published:** 2021-08-25

**Authors:** Haruna Takahashi, Sho Fukaya, Chihong Song, Kazuyoshi Murata, Masaharu Takemura

**Affiliations:** a Laboratory of Biology, Graduate School of Mathematics and Science Education, Tokyo University of Science, Shinjuku, Tokyo, Japan; b Laboratory of Biology, Institute of Arts and Sciences, Tokyo University of Science, Shinjuku, Tokyo, Japan; c Department of Applied Information Engineering, Faculty of Engineering, Suwa University of Science, Chino, Nagano, Japan; d Exploratory Research Center on Life and Living Systems, National Institutes of Natural Sciences, Okazaki, Aichi, Japan; e National Institute for Physiological Sciences, National Institutes of Natural Sciences, Okazaki, Aichi, Japan; University of Illinois at Urbana Champaign

**Keywords:** *Cotonvirus japonicus*, family *Mimiviridae*, giant virus, isolation

## Abstract

Since 2003, various viruses from the subfamily *Megavirinae* in the family *Mimiviridae* have been isolated worldwide, including icosahedral mimiviruses and tailed tupanviruses. To date, the evolutionary relationship between tailed and nontailed mimiviruses has not been elucidated. Here, we present the genomic and morphological features of a newly isolated giant virus, *Cotonvirus japonicus* (cotonvirus), belonging to the family *Mimiviridae.* It contains a linear double-stranded DNA molecule of 1.47 Mb, the largest among the reported viruses in the subfamily *Megavirinae*, excluding tupanviruses. Among its 1,306 predicted open reading frames, 1,149 (88.0%) were homologous to those of the family *Mimiviridae*. Several nucleocytoplasmic large DNA virus (NCLDV) core genes, aminoacyl-tRNA synthetase genes, and the host specificity of cotonvirus were highly similar to those of *Mimiviridae* lineages A, B, and C; however, lineage A was slightly closer to cotonvirus than the others were. Moreover, based on its genome size, the presence of two copies of 18S rRNA-like sequences, and the period of its infection cycle, cotonvirus is the most similar to the tupanviruses among the icosahedral mimiviruses. Interestingly, the cotonvirus utilizes Golgi apparatus-like vesicles for virion factory (VF) formation. Overall, we showed that cotonvirus is a novel lineage of the subfamily *Megavirinae*. Our findings support the diversity of icosahedral mimiviruses and provide mechanistic insights into the replication, VF formation, and evolution of the subfamily *Megavirinae*.

**IMPORTANCE** We have isolated a new virus of an independent lineage belonging to the family *Mimiviridae*, subfamily *Megavirinae*, from the fresh water of a canal in Japan, named *Cotonvirus*. In a proteomic tree, this new nucleocytoplasmic large DNA virus (NCLDV) is phylogenetically placed at the root of three lineages of the subfamily *Megavirinae*—lineages A (mimivirus), B (moumouvirus), and C (megavirus). Multiple genomic and phenotypic features of cotonvirus are more similar to those of tupanviruses than to those of the A, B, or C lineages, and other genomic features, while the host specificity of cotonvirus is more similar to those of the latter than of the former. These results suggest that cotonvirus is a unique virus that has chimeric features of existing viruses of *Megavirinae* and uses Golgi apparatus-like vesicles of the host cells for virion factory (VF) formation. Thus, cotonvirus can provide novel insights into the evolution of mimiviruses and the underlying mechanisms of VF formation.

## INTRODUCTION

The family *Mimiviridae* belongs to the phylum *Nucleocytoviricota*, whose members include nucleocytoplasmic large DNA viruses (NCLDVs) (1–3). Since the discovery of Acanthamoeba polyphaga *mimivirus* (APMV) (1, 2) in 2003, giant viruses, which belong to NCLDVs and include the families *Mimiviridae* (1–5) and *Marseilleviridae* (6), pandoraviruses (7), pithoviruses (8), molliviruses (9), faustoviruses (10), and medusavirus (11) have been isolated globally, and their morphological and genomic characteristics have been investigated.

The morphologies and genomes of giant viruses are diverse among families and species. In particular, *Mimiviridae* is one of the most diverse families of eukaryotic viruses (1–5, 12, 13); its members have mostly been isolated from the aquatic environment and, more recently, from soils, suggesting that they are ubiquitous (12–14). These viruses contain several complex biological systems, such as the mimivirus virophage resistance element (MIMIVIRE) (15) and many genes responsible for protein synthesis, including aminoacyl-tRNA synthetase (aa-RS) genes (1–5). Notably, members of the family *Mimiviridae* can build a cytoplasmic, organelle-like virion factory (VF) in host cells (16–18). The VF contains large amounts of replicating mimivirus genomic DNA, and new virions appear on the surface of the VF, which contains materials for capsids and inner membranes. The mimivirus VF is proposed to be localized close to the endoplasmic reticulum (ER) and derive its inner membranes from the ER or from ER-related vesicles (16–18).

The family *Mimiviridae* has been proposed to include the subfamilies *Megavirinae* (or *Megamimivirinae*), *Mesomimivirinae*, and *Klosneuvirinae* (3), which have not yet been listed by the International Committee for Taxonomy of Viruses (ICTV). *Megavirinae* is divided into four lineages, namely, lineages A, B, and C and tupanviruses, according to the results of phylogenetic analysis based on NCLDV core genes, including those of B family DNA polymerase and the major capsid protein (19). Viruses of lineages A to C have icosahedral morphology, whereas tupanviruses have a tail (1–5, 19). In addition, only the tupanviruses can infect *Vermamoeba* spp. and *Acanthamoeba* spp. (19–21). Tupanviruses also possess the largest genome and the highest number of genes among all four lineages (19, 20). However, the evolutionary relationship between tailed tupanviruses and other, nontailed mimivirus lineages of the subfamily *Megavirinae* has not been elucidated.

Here, we present a new virus, *Cotonvirus japonicus* (cotonvirus), which belongs to the subfamily *Megavirinae* but not to any of the known four lineages; rather, it has chimeric features of all four lineages. Furthermore, it was interesting to note that the cotonvirus VF is constructed in Golgi apparatus-like vesicles, not in the neighboring ER, as is the case in other mimiviruses. These newly observed features of cotonvirus provide new insights into the evolution of the subfamily *Megavirinae* and its replication mechanisms.

## RESULTS

### Isolation of cotonvirus.

Upon coculture with Acanthamoeba castellanii, a cytopathic effect (CPE) was observed. After isolation, we obtained three new viruses of the subfamily *Megavirinae*, including a novel virus (named cotonvirus herein) from Japan’s Chiba Prefecture, an unidentified virus belonging to lineage A, and a mimivirus belonging to lineage C (identified as *Megavirus musashi*) from Japan’s Saitama Prefecture; this was elucidated based on molecular phylogenetic analyses using B family DNA polymerase genes, as described below.

### Morphological features.

**FIG 1:**
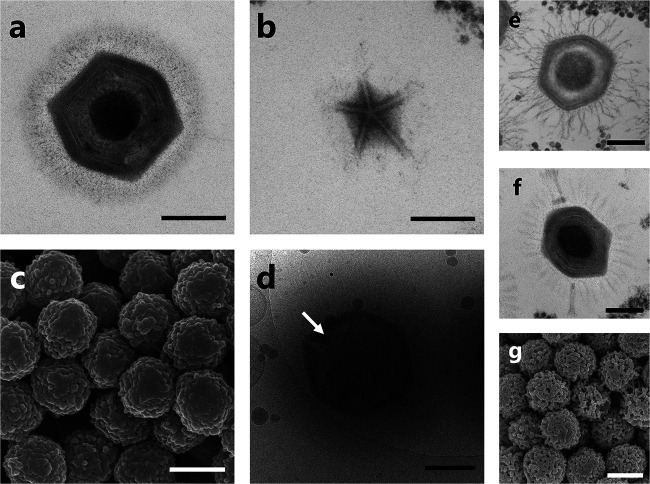
Electron micrographs of cotonvirus particles. (a, b) TEM images of a cotonvirus particle (a) and its stargate structure (b). (c) SEM image. (d) cryo-EM image showing an open stargate structure (white arrow). (e, f) TEM image of *Mimivirus shirakomae* (lineage A) (e) and *Megavirus musashi* (lineage C) particles (f). (g) cryo-EM image of *Mimivirus shirakomae* particles. Scale bars: black, 200 nm; white, 500 nm.

Transmission electron microscopy (TEM) and cryoelectron microscopy (cryo-EM) revealed that cotonvirus particles were morphologically very similar to other lineage A, B, and C mimiviruses, i.e., with three capsid layers, an inner membrane surrounding a core that stained densely, and surface fibrils (Fig. 1a and c). The cotonvirus particles exhibited an icosahedral capsid (approximately 400 nm in diameter), surface fibrils (approximately 100 nm), and a stargate structure, which is present at a single vertex of the particle for releasing the genome into the host cell, similar to those of other mimiviruses (Fig. 1a to d). The surface fibrils of the cotonvirus were denser and shorter than those of other mimiviruses (Fig. 1a, c, e, and g). Scanning electron microscope images revealed that the surface of cotonvirus particles was smoother than that of lineage A *Mimivirus shirakomae* particles (Fig. 1c and g). Although the likelihood of interference in fiber visualization by electron microscope sample preparation procedures cannot be excluded, we named the new virus *Cotonvirus japonicus* because of the cotton-like appearance of the surface fibrils (Fig. 1a).

### Host specificity.

**FIG 2:**
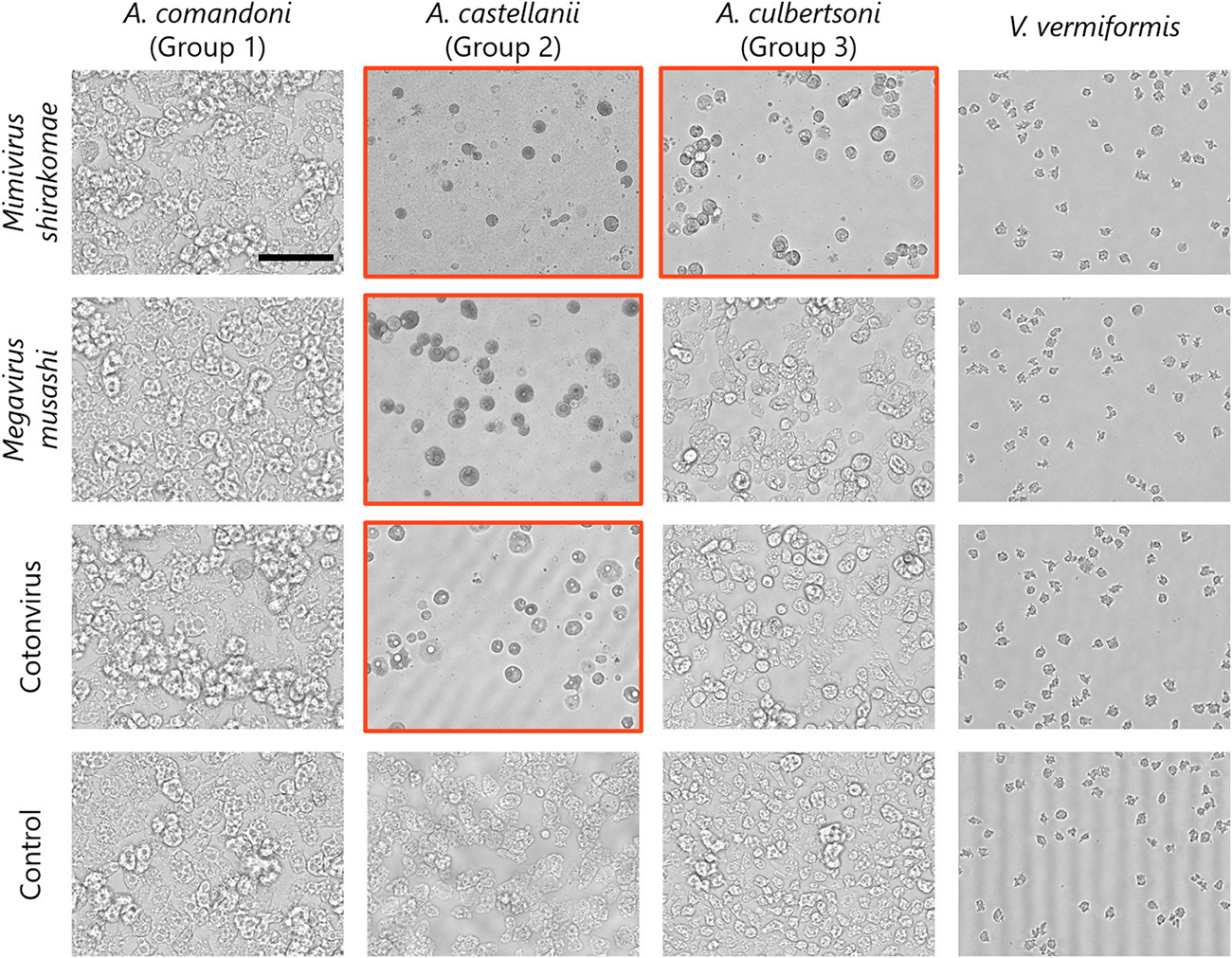
Host specificity of cotonvirus. Acanthamoeba comandoni, A. castellanii, A. culbertsoni, and Vermamoeba vermiformis cells were infected with cotonvirus, mimivirus (*Mimivirus shirakomae*), and megavirus (*Megavirus musashi*) at an MOI of 100. After 1 day, amoeba cells were observed using phase-contrast microscopy. Images in which cytopathic effect (CPE) of amoeba cells is shown are indicated by red squares. Noninfected amoeba cells were used as the control. Scale bar = 100 μm.

The genus *Acanthamoeba* is classified into three groups, namely, groups 1 to 3, based on the characteristics of their cysts (22). A. castellanii belongs to group 2, along with A. polyphaga (22). To identify cotonvirus host specificity, it was allowed to infect acanthamoeba cells of typical species of each group: Acanthamoeba comandoni (group 1), A. castellanii (group 2), and Acanthamoeba culbertsoni (group 3), respectively. All the viruses tested infected A. castellanii, but only the lineage A mimivirus infected A. culbertsoni (Fig. 2 and Table 1). Other giant viruses, including cotonvirus, lineage C (megavirus) viruses, members of the family *Marseilleviridae*, and medusavirus, did not infect A. comandoni and A. culbertsoni, suggesting that the host specificity of cotonvirus is similar to that of lineage C viruses (we did not test for lineage B). Conversely, Vermamoeba vermiformis, which has already been reported to be infected by tupanviruses (19), was not infected with cotonvirus (Fig. 2 and Table 1).

**TABLE 1:**
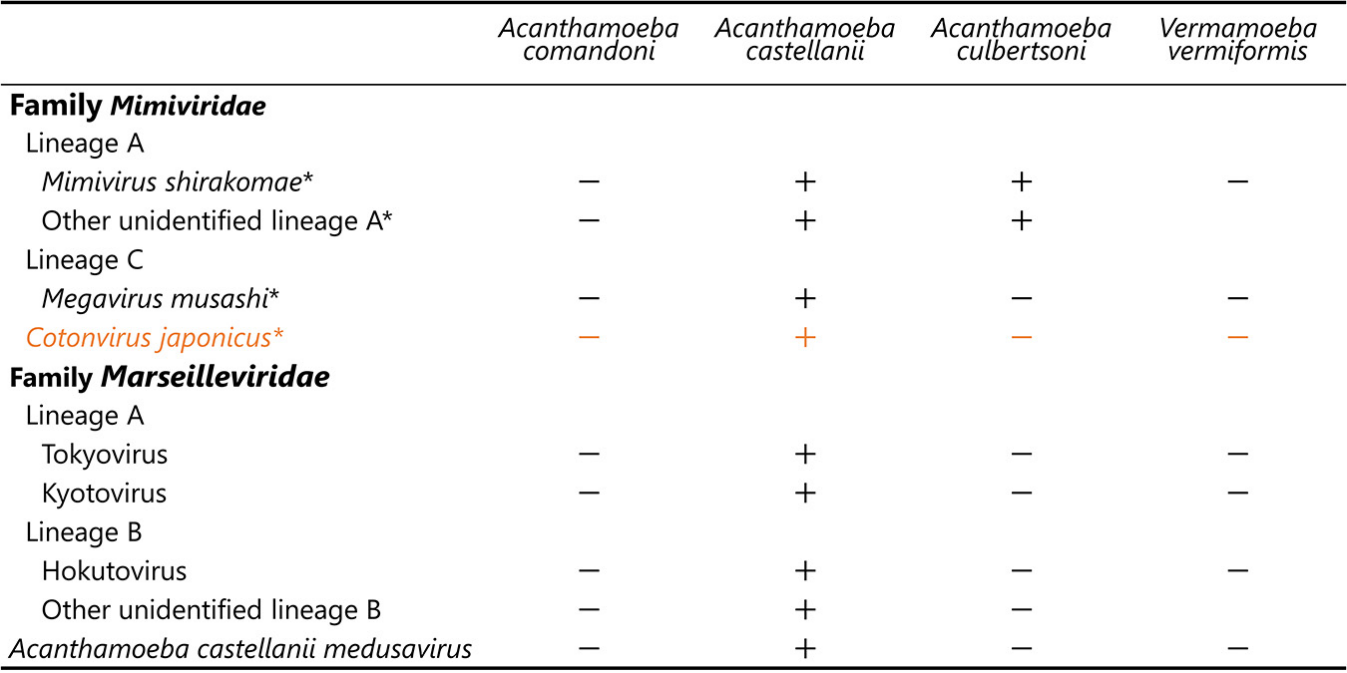
Host specificity of cotonvirus and other viruses^*a*^

a CPE was (+) observed or (−) not observed. *, these viruses infected amoeba at an MOI of 100.

### Genome.

**FIG 3:**
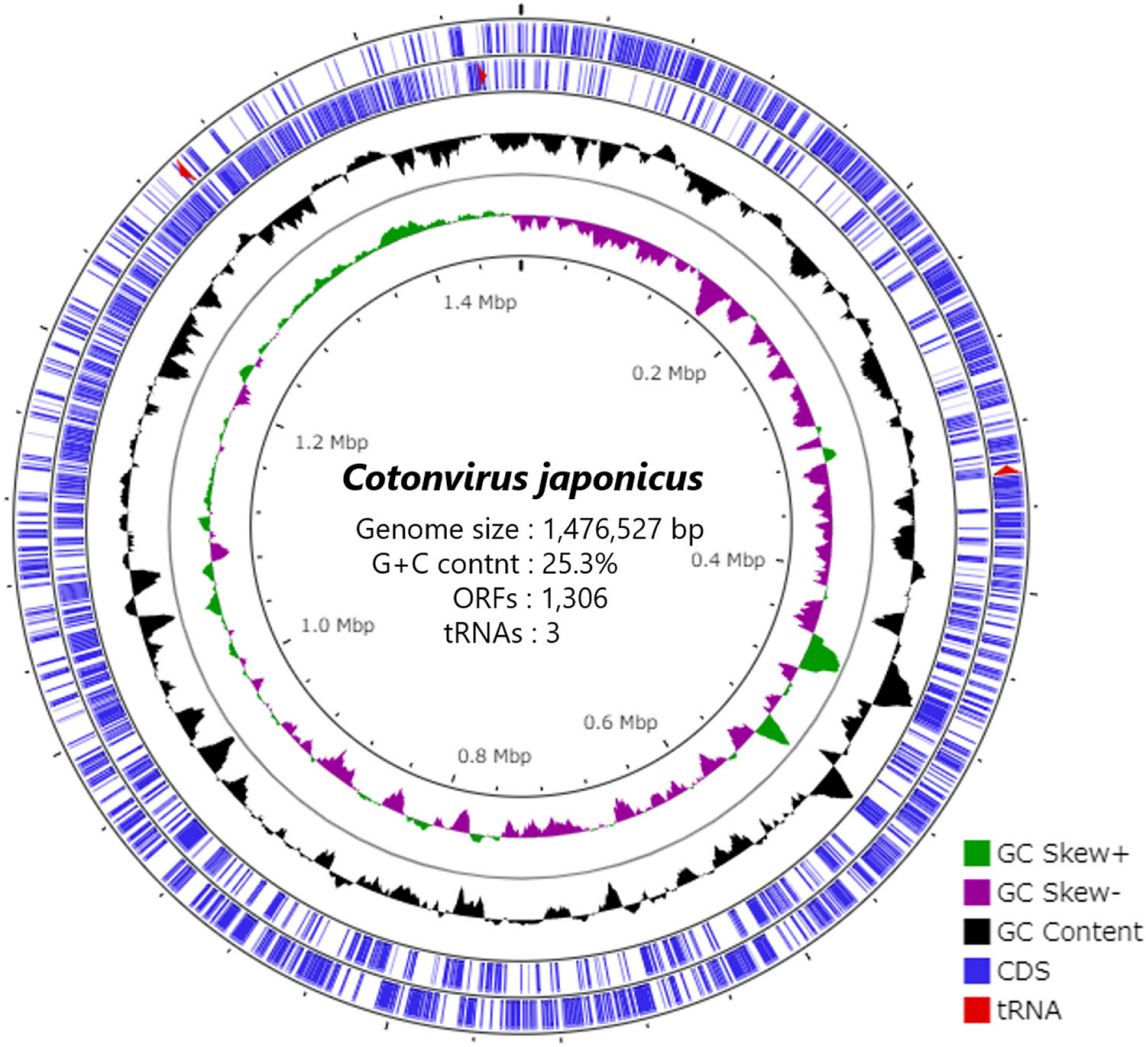
Circular representation of the cotonvirus genome. From the outside in, coding sequences (CDS) (blue) and tRNA (red), GC content (black), and GC skews (green and purple) are represented.

The cotonvirus genome is 1,476,527 bp. It is larger than those of lineages A to C of the subfamily *Megavirinae* and closer to the *Tupanvirus soda lake* (1,516,267 bp) and *Tupanvirus deep ocean* (1,439,508 bp) strains in terms of size (Fig. 3 and Table 2). In addition, the G+C content of the cotonvirus genome was 25.3%, similar to those of the lineage B moumouvirus (24.6%) and lineage C *Megavirus chilensis* (25.2%).

**TABLE 2:**
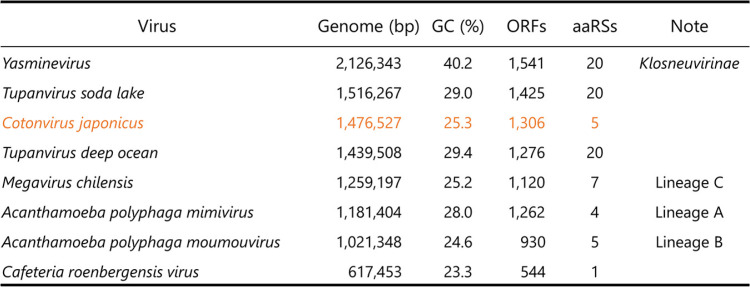
Genomic features of the cotonvirus and viruses belonging to the family *Mimiviridae*

**FIG 4:**
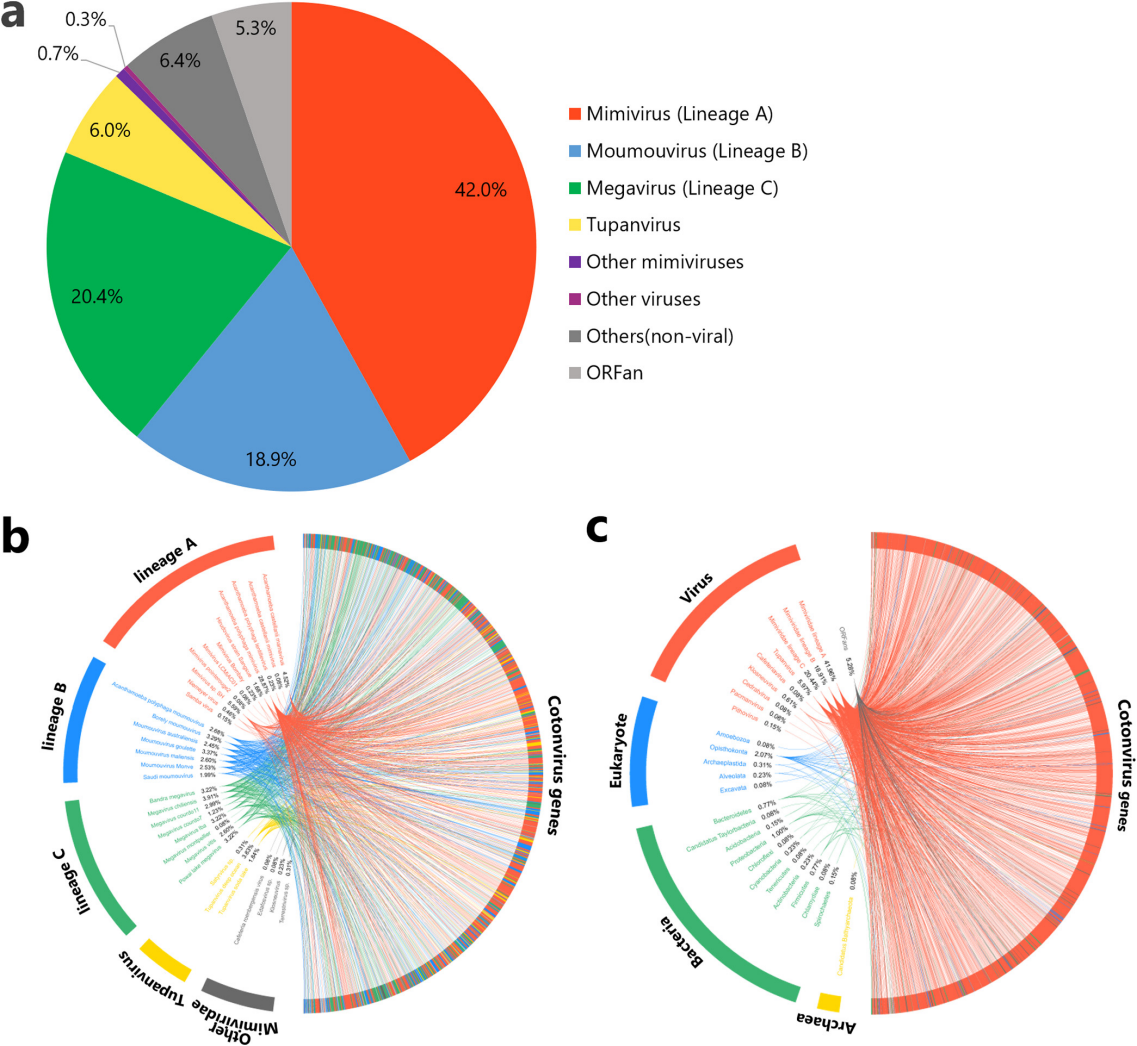
Best hits for predicted ORFs of cotonvirus. (a) Pie chart showing the best hits for amino acid homology between cotonvirus and the public sequence database. (b, c) The rhizomes show the relationship between cotonvirus genes and best hits in the family *Mimiviridae* (b) and among all species, including both living things and viruses (c).

We identified 1,306 predicted open reading frames (ORFs) (Data Set S1) and three tRNA-like sequences for tryptophan (CCA), cysteine (GCA), and leucine (TAA). The predicted number of ORFs is also similar to those of the tupanvirus soda lake and tupanvirus deep ocean strains (1,425 and 1,276, respectively) (Table 2). Among all the ORFs, 1,149 (88.0%), including those encoding annotated hypothetical proteins, showed high homology to the genes of the family *Mimiviridae* (Fig. 4a and b). The other best hits included 4 ORFs with homology to those of other viruses, 35 with homology to those of eukaryotes, 48 with homology to those of bacteria, and 1 with homology to those of archaea. A total of 69 were orphan genes (ORFans) (Fig. 4a and c). Among the ORFs that matched *Mimiviridae*, the best hits were from lineages A (42.0%), B (18.9%), and C (20.4%), tupanviruses (6.0%), klosneuviruses (0.6%), and *Cafeteria roenbergensis virus* (0.1%), suggesting that the genes of cotonvirus are homologous to the genes of the entire *Mimiviridae* family, rather than those of any particular lineages or viruses.

**FIG 5:**
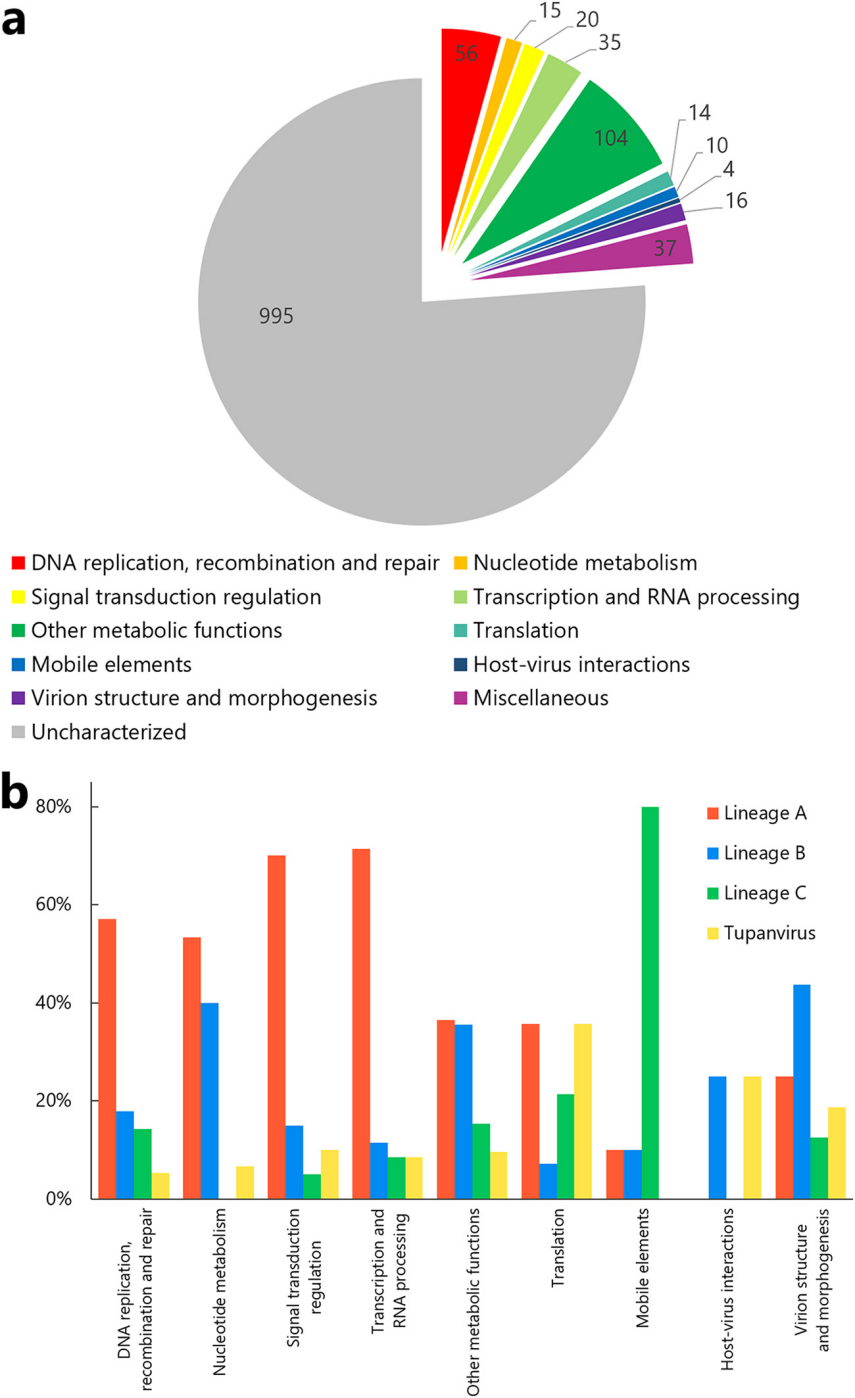
Analysis of gene categories of cotonvirus. (a) Classification of cotonvirus genes based on the functional categories of genes. (b) Proportion of each functional category of genes in the subfamily *Megavirinae* that are best hits to cotonvirus.

Moreover, we classified the cotonvirus genes based on their putative functions in functional categories (Fig. 5a). Overall, 311 (23.8%) have known functions, including “DNA replication, recombination and repair” and “other metabolic functions,” whereas 995 are classified as “uncharacterized genes,” including ORFans and hypothetical proteins; 15 genes can be classified in “translation” (Fig. 5a). Cotonvirus encodes 5 aminoacyl-tRNA synthetases (aa-RSs) (Arg-RS, Cys-RS, Ile-RS, Met-RS, and Tyr-RS) related to the translation machinery, similar to other viruses of the subfamily *Megavirinae*.

Furthermore, we calculated the proportion of each category of the genes in the subfamily *Megavirinae* that were best hits for cotonvirus genes (Fig. 5b). Among these categories, cotonvirus genes are similar to those of lineage A in the categories “DNA replication, recombination and repair,” “signal transduction regulation,” and “transcription and RNA processing.” The cotonvirus genes under the category “mobile elements” showed the greatest similarity with those of lineage C, whereas those under “virion structure and morphogenesis” were more similar to those of lineage B than to those of other lineages. In the “host-virus interaction” category, similarities among cotonvirus, lineage B viruses, and tupanvirus were observed.

These results suggest that cotonvirus is a new member and that it exhibits chimeric features of the subfamily *Megavirinae*. Our findings clearly demonstrate that cotonvirus is a novel independent lineage under *Megavirinae*, which includes icosahedral mimiviruses and tailed tupanviruses.

### Molecular phylogenetic analysis.

**FIG 6:**
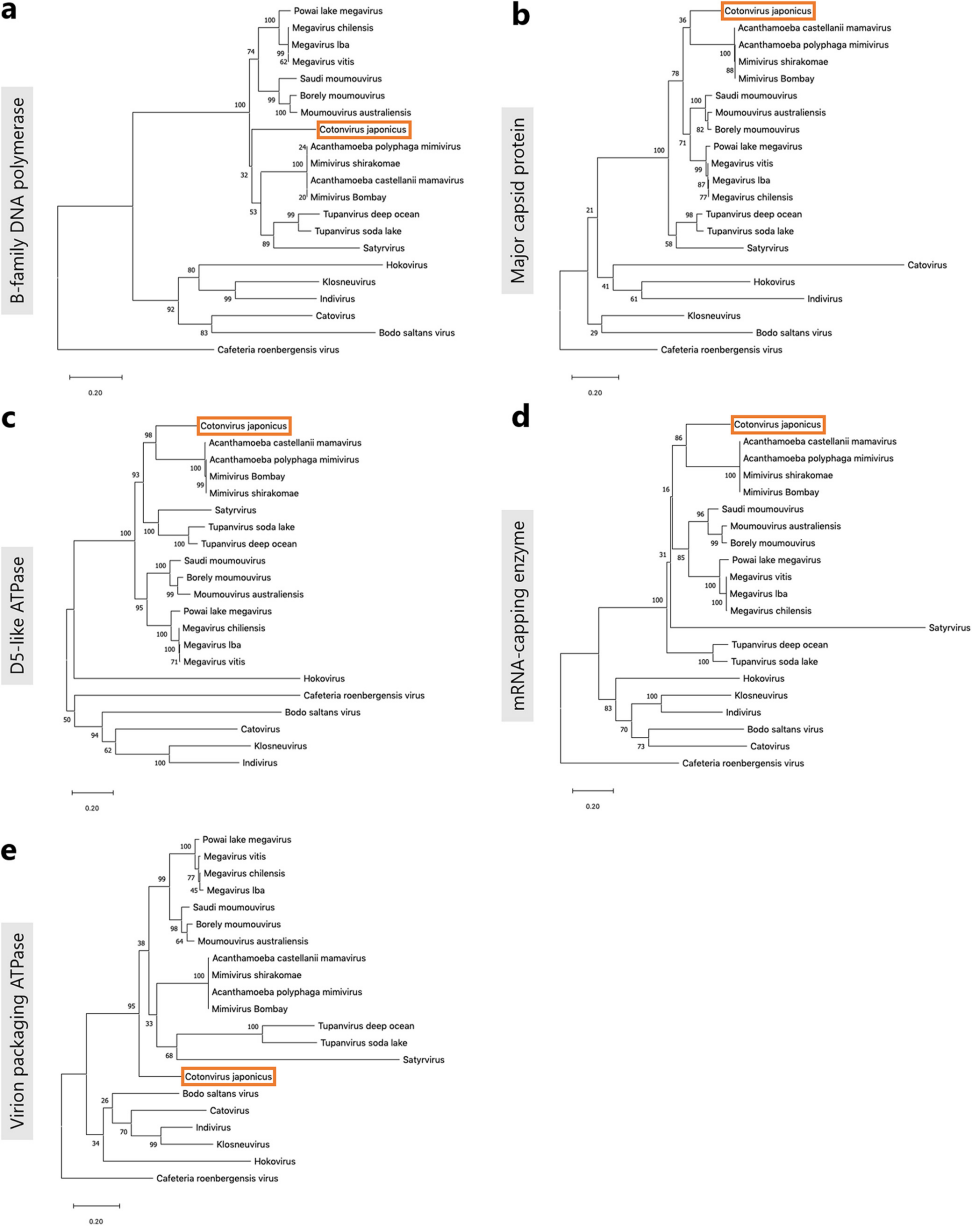
Molecular phylogenetic analysis based on the nucleotide sequences of the nucleocytoplasmic large DNA virus (NCLDV) core genes, namely, B family DNA polymerase (a), major capsid protein (b), D5-like ATPase (c), mRNA-packaging enzyme (d), and virion-packaging ATPase (e).

To estimate the phylogenetic position of cotonvirus in the subfamily *Megavirinae*, we performed molecular phylogenetic analysis based on NCLDV core genes, including those encoding B family DNA polymerase, major capsid protein, D5-like ATPase, mRNA-capping enzyme, and virion packaging ATPase, and reconstructed their molecular phylogenetic trees (Fig. 6). Cotonvirus did not belong to any known lineage and was not included in existing sister groups in each of these phylogenetic trees. In a molecular phylogenetic tree based on the B family DNA polymerase gene, cotonvirus formed a new sister group with the clade comprising lineage A and tupanvirus (Fig. 6a), whereas in one based on the major capsid protein, D5-like ATPase, and mRNA-capping enzyme genes, cotonvirus formed a new sister group with the clade comprising lineage A only (Fig. 6b). Additionally, in a molecular phylogenetic tree based on the virion-packaging ATPase gene, cotonvirus formed a sister group with the whole subfamily *Megavirinae*. These results suggest that each cotonvirus NCLDV core gene is similar to those of different lineages, indicating that cotonvirus and the other existing lineages of the subfamily *Megavirinae* diverged at different times during their evolution.

**FIG 7:**
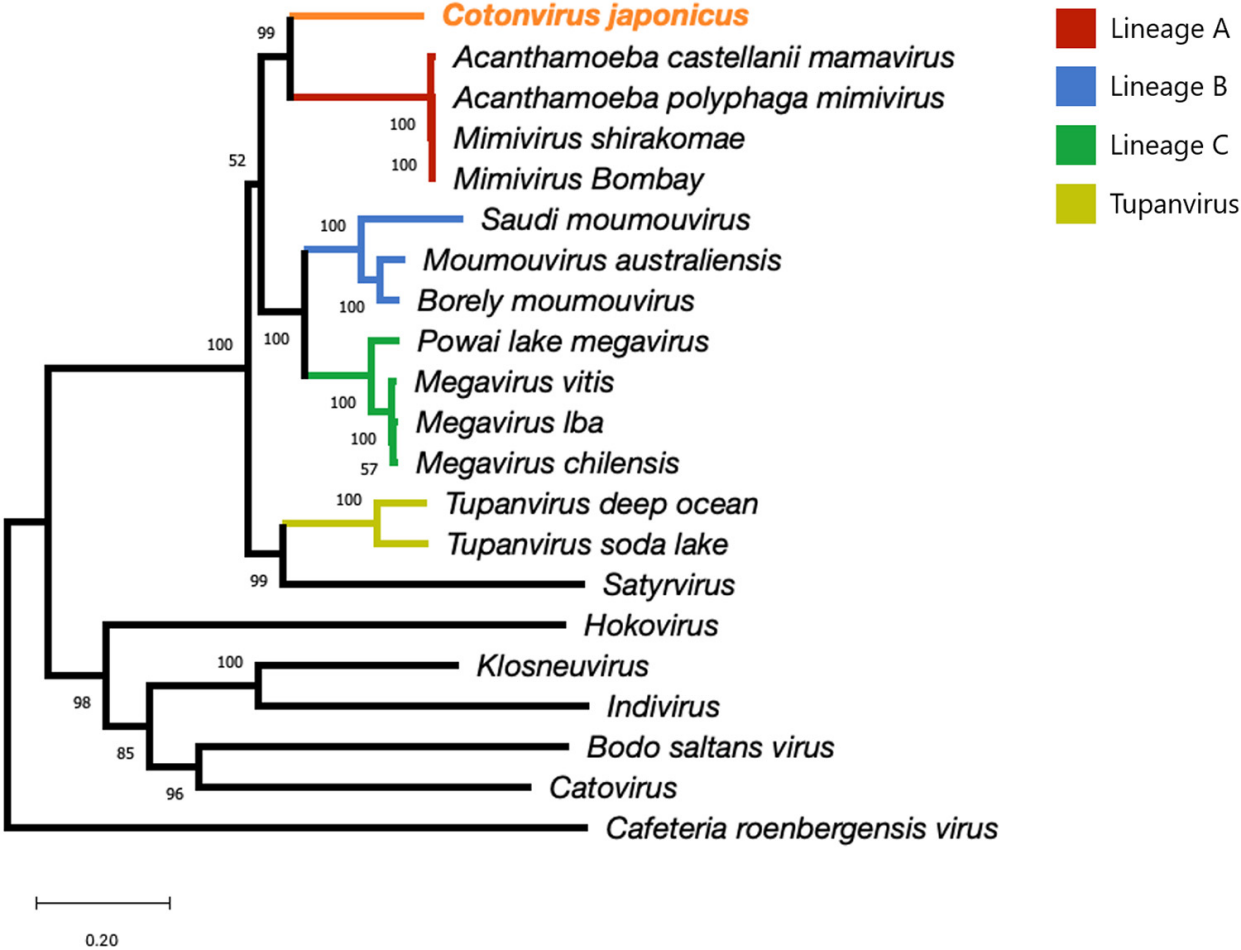
Molecular phylogenetic analysis based on the concatenated NCLDV core genes, consisting of B family DNA polymerase, major capsid protein, D5-like ATPase, mRNA-packaging enzyme, and virion-packaging ATPase.

**FIG 8:**
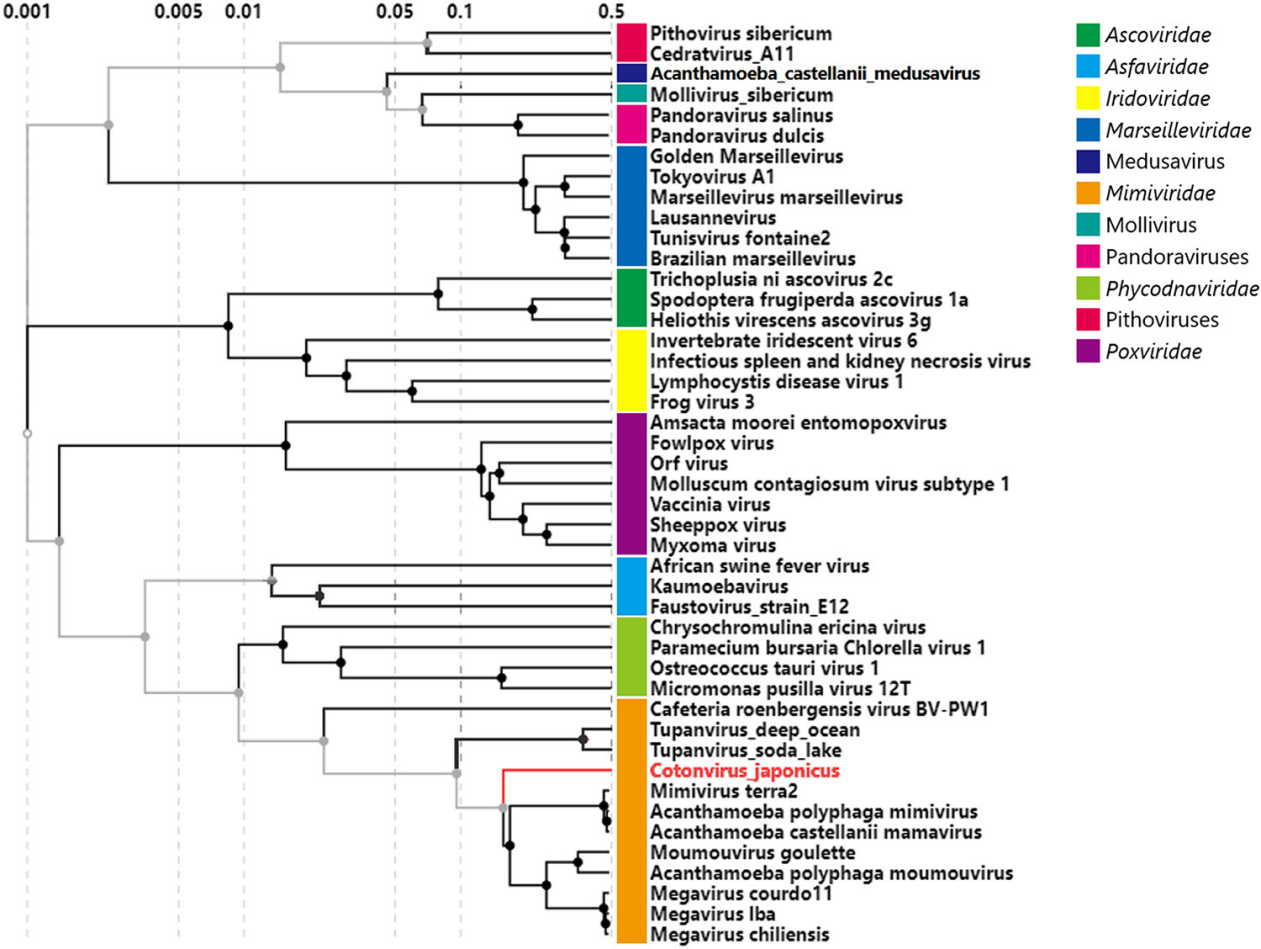
Proteomic tree based on the genomic sequences of NCLDVs. Each color represents the family or group of NCLDVs. The cotonvirus is in red.

Furthermore, based on a concatenated gene sequence consisting of the five NCLDV core genes, the cotonvirus formed a sister group with lineage A (Fig. 7). Thus, cotonvirus is phylogenetically and evolutionarily related to lineage A. According to the constructed proteomic tree, the cotonvirus branched from the root of the clade consisting of 3 lineages (A, B, and C), and these viruses, along with cotonvirus, formed a sister group with the clade comprising tupanvirus (Fig. 8). These results suggest that cotonvirus represents an independent lineage under the subfamily *Megavirinae*.

**FIG 9:**
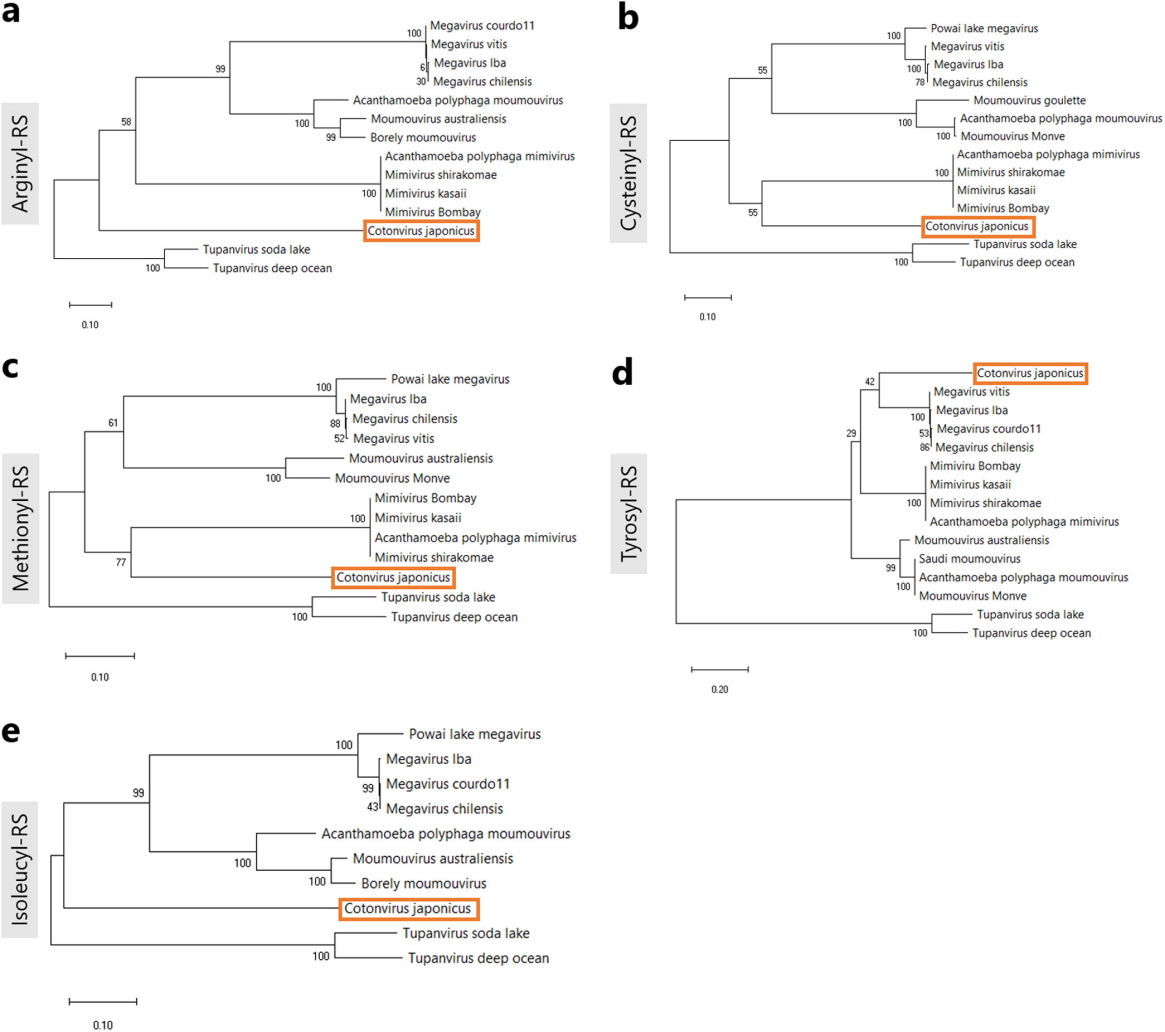
Molecular phylogenetic analysis based on the nucleotide sequences of the aa-RS genes, namely, arginyl-RS (a), cysteinyl-RS (b), methionyl-RS (c), tyrosyl-RS (d), and isoleucyl-RS (e).

Additionally, we performed molecular phylogenetic analyses based on the aa-RS genes (Fig. 9). Similar to the results of phylogenetic analyses based on the NCLDV core gene, cotonvirus was found not to belong to any existing lineage and to instead form an independent lineage. The arginyl-RS and isoleucyl-RS genes of cotonvirus represent independent lineages in the clade comprising lineages A to C (Fig. 9a and e). The cysteinyl-RS and methionyl-RS genes of cotonvirus formed sister groups with those of lineage A (Fig. 9b and c), whereas the tyrosyl-RS gene of cotonvirus formed a sister group with that of lineage C, although the bootstrap support was relatively poor (Fig. 9d). Based on these results, we concluded that molecular phylogenetic analysis has provided clues to elucidate the evolutionary perspective of the subfamily *Megavirinae*, including cotonvirus.

**FIG 10:**
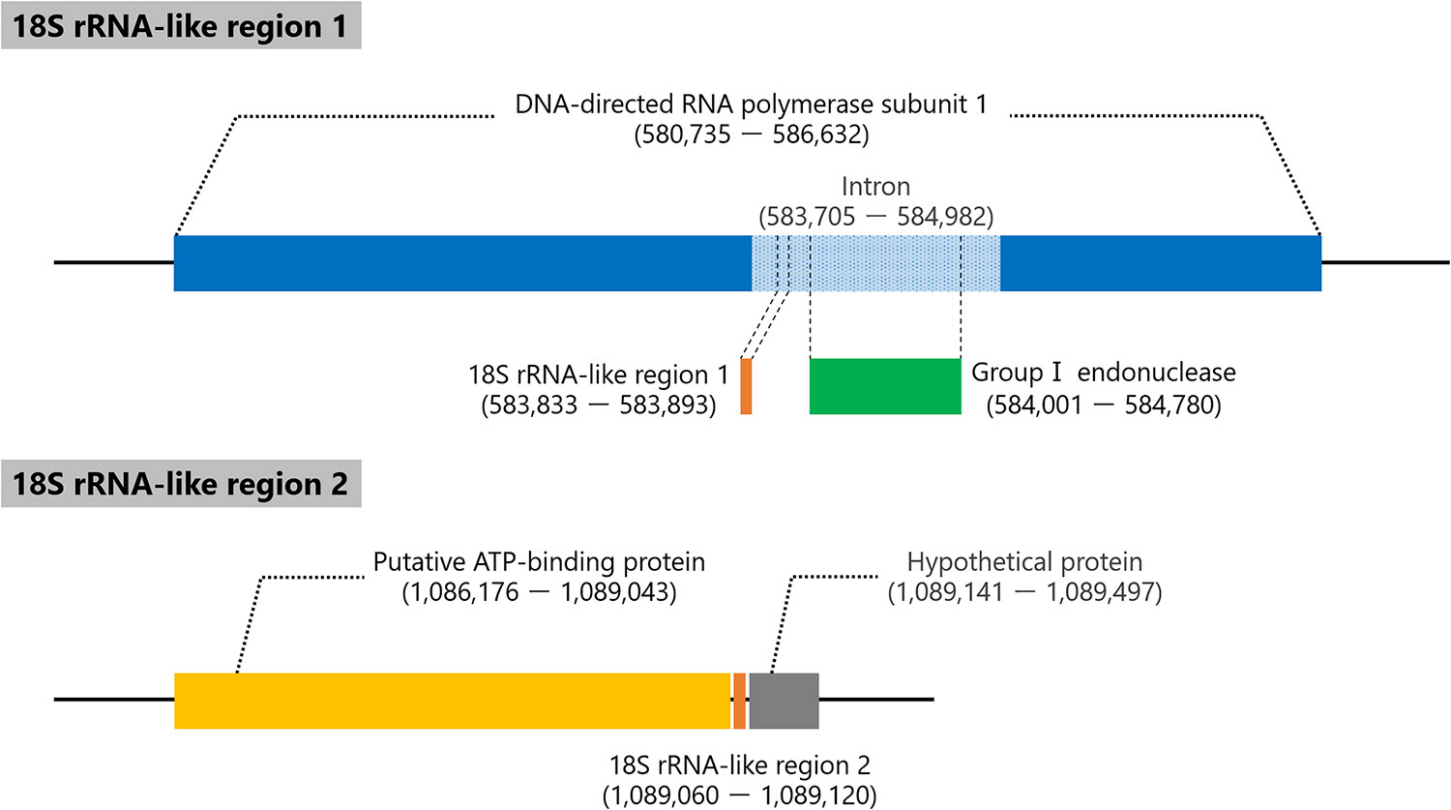
18S rRNA-like sequences of cotonvirus.

Furthermore, the 18S rRNA intronic region is widely observed in the family *Mimiviridae*, and there have been reports of there being two copies of 18S rRNA intronic regions in tupanviruses, *Borely moumouvirus* (lineage B), and lineage C viruses, whereas there is only one copy in other lineages (19, 23). In the cotonvirus genome, we detected two copies of 18S rRNA-like sequences, similar to tupanviruses and lineage C (Fig. 10), one of which exists in the intron of the DNA-directed RNA polymerase gene, similar to lineages A to C (19), whereas the other is in the neighboring region of the putative ATP-binding protein, unlike tupanviruses and lineage C viruses, whose 18S rRNA-like sequence is close to the capsid protein 1 gene (19). These data support the hypothesis that cotonvirus represents an independent lineage of the subfamily *Megavirinae*.

**FIG 11:**
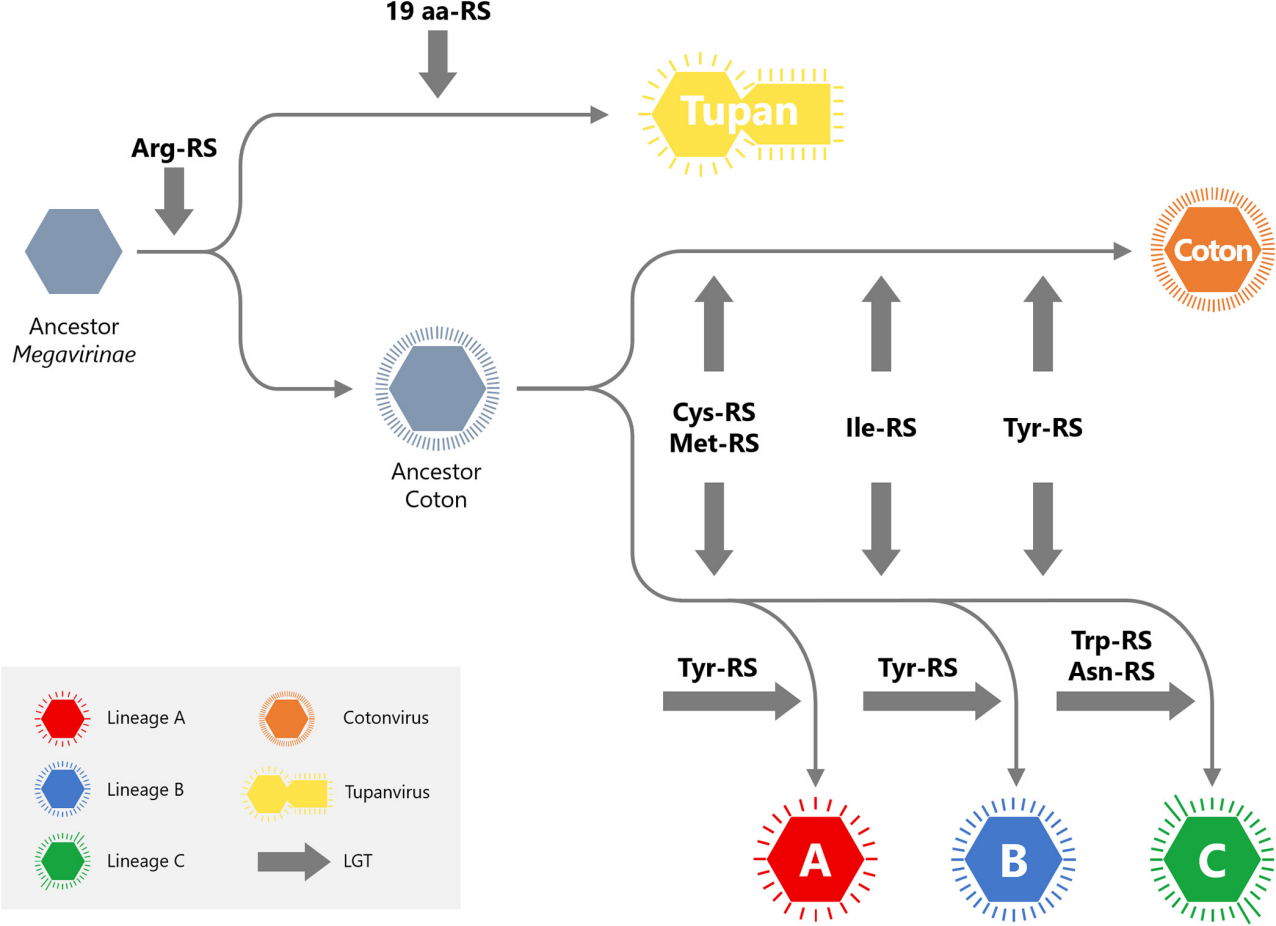
Putative evolutionary model of the subfamily *Megavirinae*. Our hypothesis is based on the molecular phylogenetic analyses of the proteomic tree and the aa-RS genes. Thick arrows (gray) indicate LGT.

Thus, we propose a putative evolutionary model for cotonvirus and for the subfamily *Megavirinae* (Fig. 11). The common ancestor of the subfamily *Megavirinae* gave rise to the ancestors of the tupanvirus and cotonvirus, and the latter further gave rise to the ancestors of cotonvirus and of lineages A, B, and C. We hypothesized that each lineage of the subfamily *Megavirinae* evolved by acquiring genes via lateral gene transfer (LGT) (24–26). The putative evolutionary model is consistent with the genomic features of cotonvirus, whose genes are highly homologous with those of lineages A to C and whose genome complexity is similar to that of tupanvirus.

### Sequences of virophages in cotonvirus genome.

We tested whether virophage sequences, such as the 28-nucleotide-long Zamilon insert sequence (AATCTGATAATGAATCTGATAATGAATC), which has been observed in reported APMV genomes (15), are present in the cotonvirus genome. We have detected this sequence in the cotonvirus genome at only one site: in a complementary strand of ORF1052 (annotated as “hypothetical protein”) at genome position 1,204,030 to 1,204,061 (data not shown). This Zamilon insert-like sequence is 32 nucleotides long (GAATCTGATAATGAATCTGATAATGAATCCGA), which is longer than that in the APMV genome (15). Conversely, we did not detect the derived 15-nucleotide repeated unit, TGATAATGAATCTGA, and Cas-like gene, as presented in the genomes of lineage A viruses. Therefore, we have not presently confirmed that there is a MIMIVIRE-like system in the cotonvirus genome as in the lineage A mimivirus genome (15). Additionally, we did not detect sequences highly homologous with those of virophages Sputnik (27) or Guarani (28) in the cotonvirus genome.

### Infection cycle by time-lapse imaging.

**FIG 12:**
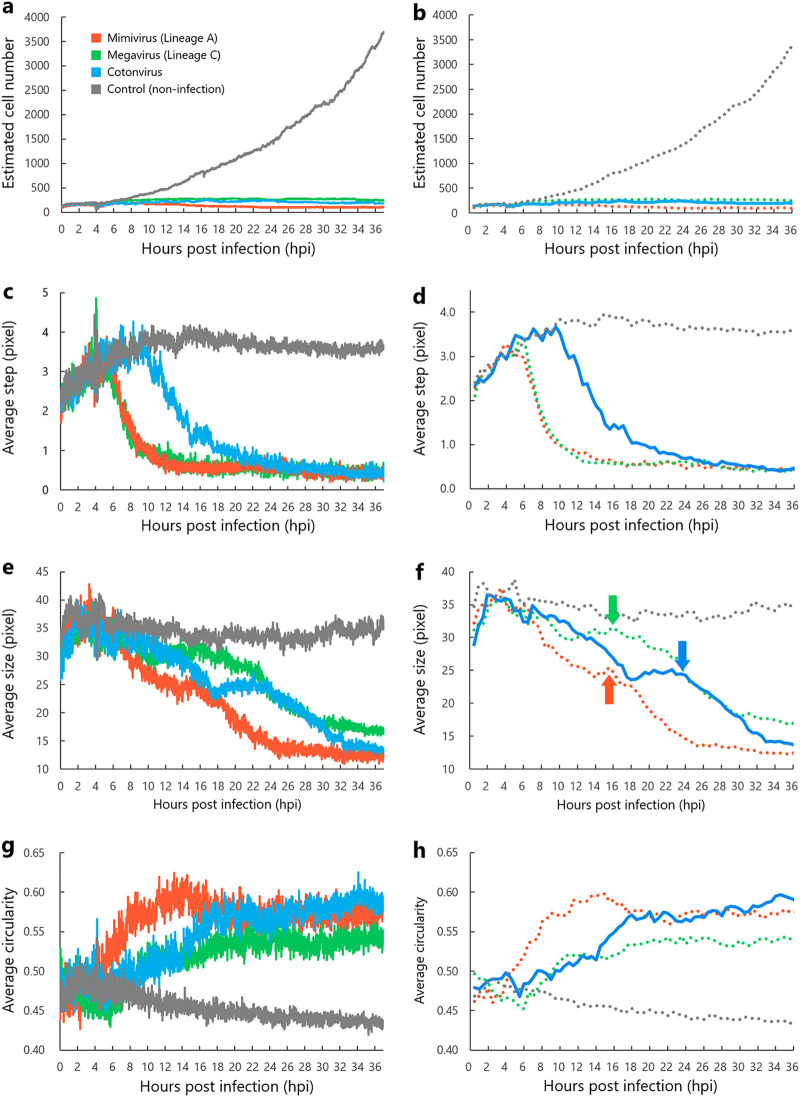
Kinetics of cotonvirus-infected A. castellanii cells analyzed by the PKA3 algorithm. We measured the estimated cell number (a, b), average step (c, d), average size (e, f), and average circularity (g, h) of cotonvirus-infected A. castellanii cells from 0 to 36 hpi. The graphs on the right show the average value every 30 min. Mimivirus (*Mimivirus shirakomae*) and megavirus (*Megavirus musashi*) were analyzed for comparison with cotonvirus. Noninfected A. castellanii cells were used as the control. (f) Red, green, and blue arrows indicate the starting points of cell lysis in each virus-infected A. castellanii cell.

Using time-lapse imaging for 36 h, we observed the kinetics of cotonvirus-infected A. castellanii cells and compared it with the kinetics of A. castellanii cells infected with *Mimivirus shirakomae* (lineage A) and *Megavirus musashi* (lineage C) (Fig. 12; Movie S1 and Supplemental Data Set and Movie Legends). Figure 12 shows graphical representations of values for each virus infection (left) and the average value of each virus infection every 30 min (right). The estimated cell numbers did not increase in mimivirus-, megavirus-, and cotonvirus-infected A. castellanii cells (Fig. 12a and b), but they finally decreased due to cell lysis at the end of the infection (data not shown). On the other hand, there were differences in cell migration (which represents the distance of movement of A. castellanii cells from one frame to the next frame of time-lapse images) among the groups. The average numbers of mimivirus- and megavirus-infected A. castellanii cells decreased after 4 to 6 h postinfection (hpi), whereas the cotonvirus-infected A. castellanii cells decreased only after 10 hpi, suggesting that the CPE of cotonvirus is expressed later than that of lineage A and C viruses (Fig. 12c and d).

Because lineage A mimivirus-infected cells could form a VF at 4 to 6 hpi (16), this suggests that the average step (average moving distance) of cotonvirus-infected A. castellanii cells delays the formation of VF. Based on these results, we estimated that cotonvirus-infected A. castellanii cells would begin to form cotonvirus VFs at 10 hpi. The average sizes of mimivirus-, megavirus-, and cotonvirus-infected A. castellanii cells decreased, plateaued, and then started to decrease again (Fig. 12e and f). These cells had a round morphology due to CPE, and thus, their sizes were smaller than the original sizes of healthy trophozoites. Subsequently, they spread the new virions via cell lysis at the end of infection, resulting in a further reduction in size. Therefore, cell lysis was considered to begin during the second decrease (Fig. 12e and f). The average size of cotonvirus-infected A. castellanii cells decreased again at 24 hpi, which was later than those infected with mimivirus and megavirus; therefore, the cotonvirus infection cycle was completed and numerous virions were released after approximately 24 hpi. Similar to the rounding, which was observed microscopically, the sphericality of the mimivirus-, megavirus-, and cotonvirus-infected A. castellanii cells gradually increased and plateaued at 10 (mimivirus and megavirus) or 18 (cotonvirus) hpi (Fig. 12g and h).

### Maturation of virion factory using host Golgi apparatus-like vesicles.

**FIG 13:**
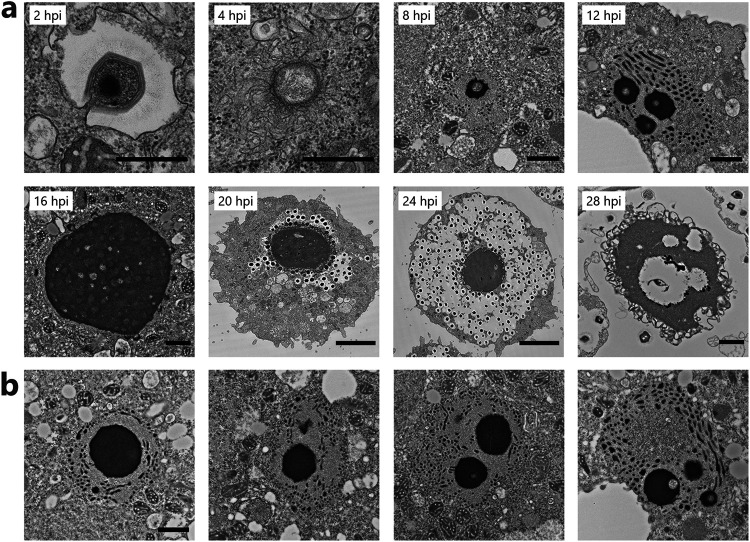
VF formation of cotonvirus. (a) Development of cotonvirus VF. First, the inner materials of cotonvirus particles are released and engulfed by amoeba cells into the host cytoplasm at 2 hpi. The cotonvirus core and membrane-like structures surrounding it are visualized at 4 hpi. Early VFs are visible at both 8 and 12 hpi, and Golgi apparatus-like structures are found around early VFs. Mature VF is formed, and the production of viral particles is initiated at 16 hpi. New virions are then produced and accumulate in the host cytoplasm at 20 hpi and 24 hpi, respectively. VF release by cell lysis is observed at 28 hpi. Scale bars: 2 hpi, 4 hpi, 500 nm; 8 hpi to 16 hpi, 28 hpi, 1 μm; 20 hpi, 24 hpi, 5 μm. (b) Four other views of early VFs surrounded by Golgi apparatus-like structures at 12 hpi.

We then investigated the formation of the VF using TEM. At 2 hpi, cotonvirus was engulfed by the phagosome of A. castellanii cells, which led to the opening of the stargate structure and the release of its inner materials into the host cytoplasm (Fig. 13a). At 4 hpi, the viral core, which appeared to be surrounded by an intertwined membrane-like structure, was observed to be released into the host cytoplasm. At 8 hpi, VF started to form in the host cytoplasm; it was fully developed at 16 hpi. Interestingly, we observed electron-dense organelle-like structures, which represent a dot- and filament-like form around the early VFs (Fig. 13a and b), that resembled the Golgi apparatus previously reported in the cysts of A. castellanii (22). At 16 and 24 hpi, new virions were assembled at the edge of the VF and accumulated in the host cytoplasm (Fig. 13a). Finally, cell debris and a piece of the VF were observed at 28 hpi, suggesting that cell lysis due to the many duplicated cotonvirus particles occurred only after 24 hpi. The duration of one cotonvirus infection cycle, from its addition to A. castellanii culture until cell lysis, is similar to that of tupanvirus (19).

**FIG 14:**
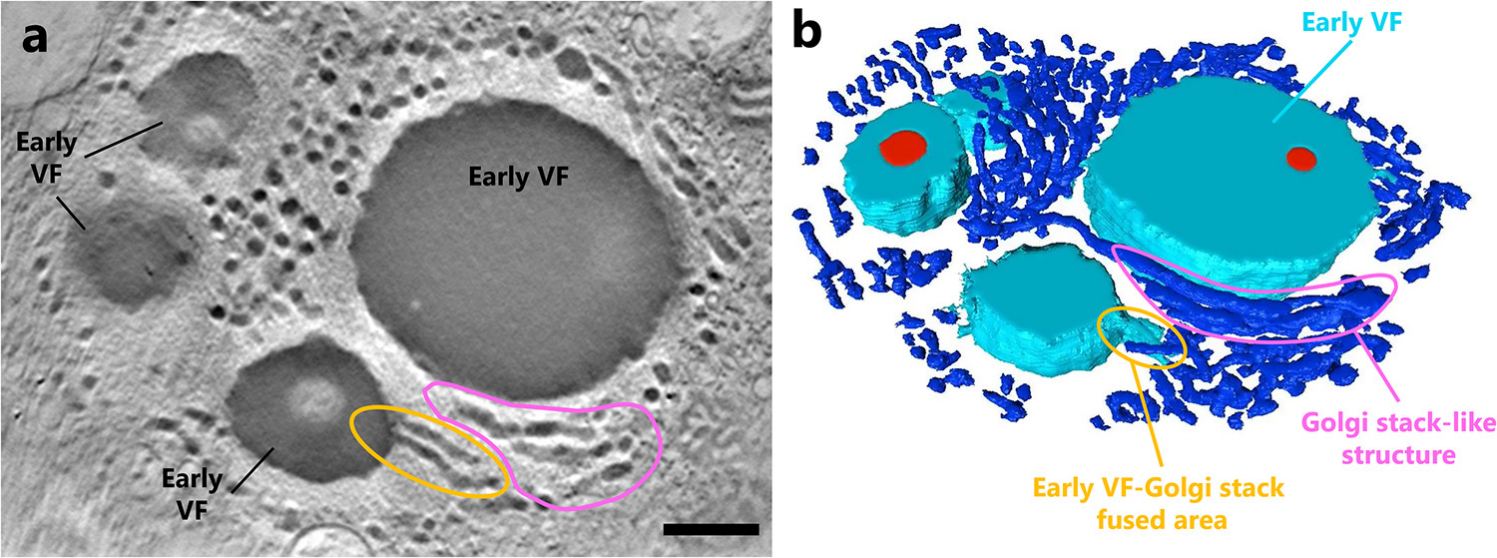
STEM tomography of the VFs in cotonvirus-infected A. castellanii. (a) Tomographic slice. (b) 3-D model. Many flat and curved tubular structures (dark blue) are observed around early VFs (light blue). The area enclosed in pink shows the layered tubular-structures similar to the Golgi stack, whereas the area enclosed in orange shows a fusion of the early VF and the tubular structures. Scale bar = 500 nm.

To depict the conformation of the Golgi-like structures around the early VF, scanning transmission electron microscopy (STEM) tomography was performed on a 12-hpi sample. We detected flat and curved tubular structures similar to the previously reported Golgi stack (22) around the early VF (Fig. 14; Movie S2). In addition, some early VFs were combined with the flat and curved tubular structures, which were then incorporated by early VFs (Fig. 14).

**FIG 15:**
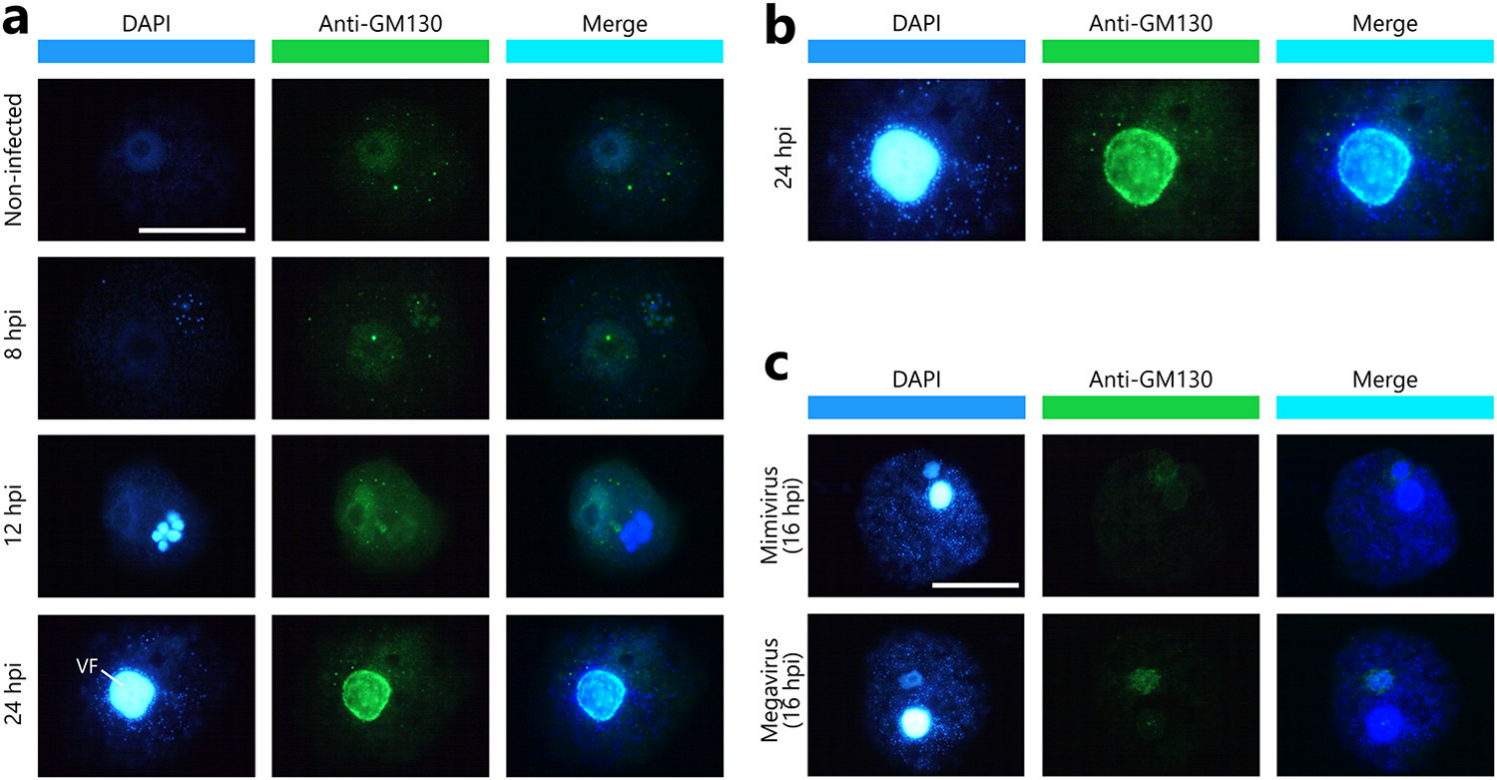
Immunofluorescent staining of A. castellanii cells infected with cotonvirus. Blue signals represent DNA in VFs, virus particles, and host nucleus stained with DAPI. Green signals represent GM130 protein of Golgi apparatus labeled by anti-GM130 antibodies. (a) A. castellanii cells infected with cotonvirus. (b) Enlarged views of images in panel a at 24 hpi. (c) A. castellanii cells infected with mimivirus (*Mimivirus shirakomae*) and megavirus (*Megavirus musashi*) at 16 hpi. Scale bar = 20 μm.

Because the cotonvirus VF was formed within Golgi apparatus-like vesicles (Fig. 12), we next performed immunofluorescence experiments using anti-GM130 antibodies, which target *cis*-Golgi matrix protein 130 (GM130) (Fig. 15). GM130 antibodies were clearly localized at the edge of matured VFs at 24 hpi compared with early VFs (Fig. 15a and b). This strong GM130 signal was not observed in the mature VFs of viruses of lineage A (mimivirus) or C (megavirus) (Fig. 15c). Thus, these results strongly suggest that the cotonvirus VF is derived from the membranes of the Golgi apparatus; this feature of VF formation has only been observed in cotonvirus-infected cells.

In APMV, it has been reported that the maturation of VF and production of viral particles require ATP and nucleotides for the synthesis of viral DNA, with VFs being surrounded by numerous mitochondria (16). In the cotonvirus infection cycle that we observed, we did not detect mitochondria surrounding VFs; therefore, in the present study, we do not confirm that cotonvirus VFs also require mitochondrial function similarly to APMV.

## DISCUSSION

In this study, we isolated three new viruses belonging to the subfamily *Megavirinae* (family *Mimiviridae*), one of which was named cotonvirus and has several unique features not observed in other existing lineages of the subfamily *Megavirinae*, including APMV, moumouvirus, and megavirus.

Genome analysis showed that cotonvirus has the largest genome (1,476,527 bp long) among the existing icosahedral lineage A, B, and C viruses, and its genome size is similar to those of tupanviruses (18). A proteomic tree reconstructed using VipTree (29) suggested that the root of the cotonvirus genome was positioned after the branching of tupanviruses and other lineages. Furthermore, the cotonvirus genome has 1,306 ORFs, including ORFans, which is close to the number of ORFs of tupanviruses. The existence of two candidate 18S rRNA-like sequences in the cotonvirus genome is also similar to what has been observed in tupanviruses. The results suggest that the overall structure of the cotonvirus genome is closer to that of the tupanvirus genome than to those of other icosahedral mimivirus genomes. Conversely, several NCLDV core genes, including those encoding major capsid protein, D5-like ATPase, and mRNA-capping enzymes, are phylogenetically closer to those of icosahedral mimivirus lineage A viruses than of tupanvirus, and host specificity trends where cotonvirus only infects A. castellanii but not V. vermiformis are consistent with the trends in icosahedral mimiviruses and not tupanvirus.

Phylogenetic analysis of individual genes, as well as the molecular phylogenetic analysis of five NCLDV core genes, revealed that cotonvirus has a slightly higher number of genes homologous with those of lineage A of the subfamily *Megavirinae* than of genes homologous with those of other lineages, including tupanviruses, in the functional categories of DNA replication, recombination, and repair, transcription and RNA processing, and signal transduction. Additionally, molecular phylogenetic analysis of 5 aa-RS genes suggests that the cotonvirus may have independently acquired these genes during the evolutionary process. Three of the five aa-RS genes of cotonvirus are more similar to those of lineage A viruses than to others, but the remaining two genes represent independent lineages. Based on these results, we propose an evolutionary scenario for cotonvirus, stating that the common ancestor of the subfamily *Megavirinae* was first divided into the ancestral tupanvirus and the ancestral cotonvirus, which then further divided into the independent cotonvirus lineage and lineages A to C.

We attribute the relatively chimeric phylogenetic relationships between the individual genes of cotonvirus and their homologous genes of other lineages to the branching before lineages A to C, which have diverged. Several independent mimiviruses that can also infect acanthamoeba cells have been reported (4), suggesting that simultaneous infection by cotonvirus and other viruses may have occurred, followed by LGT between cotonvirus and other lineages that share their hosts.

Notably, we did not observe MIMIVIRE-like repeat sequences similar to that in APMV, but we did observe one 32-nucleotide-long Zamilon insert-like sequence in the complementary strand of ORF1052 in the cotonvirus genome. This suggests that an LGT between cotonvirus and Zamilon occurred in the past, and if it implies that cotonvirus has just recently acquired the defense against Zamilon, its molecular mechanism is distinct from that in APMV. Otherwise, we may be observing the evolution of the cotonvirus’ defense system in action. Although we did not detect any MIMIVIRE-like systems similar to that in Zamilon in the present study, it is still possible that cotonvirus has unknown systems similar to MIMIVIRE. In future analyses, the infectivity of virophages like Zamilon, Sputnik, and Guarani in cotonvirus should be investigated.

The cotonvirus infection cycle was investigated in detail by analyzing the kinetics of cotonvirus-infected A. castellanii cells and VF formation using various microscopes. The PKA3 software recently developed in our laboratory can reveal the giant-virus-infected A. castellanii cell-specific motilities using time-lapse imaging under phase-contrast microscopy (30). Using this algorithm, we estimated two time points of note in the cotonvirus infection cycle: the starting point of VF formation (approximately 10 hpi) and the endpoint of the infection cycle, which represents the cell lysis of virus-infected A. castellanii cells (>24 hpi). In combination with TEM analysis, what occurs at these time points can be explained based on their actual intracellular status.

Interestingly, TEM and STEM analysis suggested that the VF formation of cotonvirus started in the Golgi apparatus-like vesicles of A. castellanii cells, which have been reported to change in morphology between trophozoite and cyst stages and to include more electron-dense materials in cysts than in trophozoites (22). These Golgi apparatus-like structures are filled with electron-dense materials typical of those observed in a cyst of A. castellanii cells, as previously reported (22), suggesting that cotonvirus infection results in a host cell response to cyst-forming conditions. Along with the observed colocalization of GM130, these observations strongly suggest that the membranes of the host Golgi apparatus are used for constructing VFs. GM130 is a peripheral membrane protein frequently found on the *cis* face of the Golgi apparatus and is involved in the sac mooring/dissociation of Golgi stack formation (31, 32). A previous study has shown that membrane assembly of mimivirus particles occurs at the edge of VFs. The inner membrane of mimivirus particles has also been suggested to be derived from the ER (16–18). However, based on our observation of the structures around early VFs and the colocalization of GM130 to the edge of matured VFs, we propose that the membrane assembly or detachment of the VFs of cotonvirus particles involves or requires the function of the membranes of the Golgi apparatus, which is different from what has been observed for other icosahedral lineages. In other words, we propose that the inner membranes of cotonviruses are directly provided by the host Golgi apparatus.

The structural differences around the core and early VFs were presumably due to the morphological changes in the host Golgi apparatus during VF formation, i.e., when the host Golgi body became encysted. The higher fluorescence intensity of anti-GM130 antibody at 24 hpi than at other time points may be due to the aggregation of scattered Golgi apparatuses in the host cytoplasm or the high expression of GM130 at the end of the infection cycle. On the other hand, we have not yet obtained evidence of the transport of some cotonvirus-encoded proteins into the Golgi apparatus, the involvement of such proteins in VF formation within the Golgi apparatus, or the recruitment of Golgi-derived inner membranes of cotonviruses for subsequent viral infection processes. Further studies are needed to determine how the functions of the host Golgi apparatus are used for virus production. In addition, because this phenomenon was not observed in mimivirus- and megavirus-infected A. castellanii cells, how GM130 is used during cotonvirus replication should be investigated.

In summary, we isolated a new lineage of the subfamily *Megavirinae* named cotonvirus, which has not previously been identified by metagenomic analysis or isolated from environmental samples. Analysis of the infection cycle and genomic features of cotonvirus provided new insights into the subfamily *Megavirinae*. Our results have important implications for understanding the replication mechanisms and evolution of the subfamily *Megavirinae* and, consequently, the family *Mimiviridae*.

## MATERIALS AND METHODS

### Virus isolation, purification, and titration.

Acanthamoeba castellanii (Douglas) strain Neff (ATCC 30010) was purchased from the American Type Culture Collection (ATCC, Manassas, VA, USA). A. castellanii cells were cultured in proteose peptone-yeast extract-glucose (PYG) medium at 26°C as previously described (7, 11, 33). Water samples (50 ml) were collected from a canal in Chiba, Japan (35°20′06.2″N, 139°50′44.2″E), and an irrigation ditch in Saitama, Japan (35°51′37.0″N, 139°48′46.7″E), and then stored at 4°C until inoculation. An aliquot (4.5 ml) was mixed with 2× PYG medium (4.5 ml) and an antibiotic solution (360 μl) as described previously (33) and added to the A. castellanii cell suspension (50 μl). The mixture was inoculated into a 96-well microplate and incubated at 26°C. After 3 to 4 days, we observed cytopathic effects (CPE) in A. castellanii cells. The supernatant was seeded into fresh A. castellanii cells, and the virus was cloned by serial dilution. The supernatant of the most diluted well that showed CPE was seeded into a fresh A. castellanii cell suspension in a 25-cm^2^ culture flask and incubated at 26°C. After 4 days, the supernatant was collected and centrifuged at 500 × *g* for 5 min to remove cellular debris. Then, the supernatant was centrifuged at 8,000 × *g* for 35 min to obtain the viral pellet. The viral pellet was washed with phosphate-buffered saline (PBS), filtered through a 1.2-μm filter (GVS Filter Technology, Inc., Indianapolis, IN, USA), and diluted in PBS. Ultimately, we isolated two new mimiviruses: *Cotonvirus japonicus* (a new lineage) from Chiba and *Megavirus musashi* (lineage C) from Saitama, which were identified using phylogenetic analysis. The viral titer was calculated using the endpoint method with TCID_50_ (50% tissue culture infective dose) Calculator version 2.1 (Marco Binder, Department of Infectious Diseases, Molecular Virology, Heidelberg University).

### Host specificity.

A. comandoni and A. culbertsoni cells gifted by Mamoru Niikura from Kyorin University were cultured in PYG medium as described above. Vermamoeba vermiformis (Hartmannella vermiformis Page; ATCC 50237) was purchased from ATCC and cultured in modified PYNFH medium. The modified PYNFH medium was prepared as follows. PYNFH medium (880 ml) was prepared by dissolving peptone (10.0 g, catalog number 211677; Gibco), yeast extract (10.0 g, catalog number 212750; BD Bioscience), RNA (1.0 g, product number R6625; Sigma-Aldrich), folic acid (15.0 mg, product number 89-10321l; WAKO), and hemin (1.0 mg, product number 060-01802; WAKO) into distilled water. Subsequently, 100 mM Na-K buffer, pH 6.5 (1 liter), was prepared using KH_2_PO_4_ and Na_2_HPO_4_. Afterward, PYNFH medium (880 ml) was mixed with 100 ml fetal bovine serum and 20 ml Na-K buffer.

Amoeba cells (A. castellanii or V. vermiformis; 2.0 × 10^4^) were mixed with PYG medium (100 μl) and viruses of the family *Mimiviridae* (MOI = 100) or other viruses, including medusavirus and those of the family *Marseilleviridae* (MOIs were not calculated). The mixture was inoculated into a 96-well microplate and incubated at 26°C. After 1 day, we determined the presence of CPE using an all-in-one fluorescence microscope (BZ-X800/X810; Keyence Co., Osaka, Japan) with a 20× objective lens.

### TEM.

A. castellanii cells were infected with the virus at a multiplicity of infection (MOI) of 100 and incubated at 26°C. After 2, 4, 8, 12, 16, 20, 24, and 28 hpi, cells were collected by centrifugation at 500 × *g* for 5 min, washed with PBS, fixed with 2% glutaraldehyde (GA), washed again with PBS, stained with 2% osmium tetroxide, and dehydrated with increasing ethanol concentrations (50%, 70%, 80%, 90%, 95%, and 100%) and propylene oxide as described previously (33). Dehydrated cells were embedded in EPON-812 resin (TAAB Laboratory Equipment, Aldermaston, United Kingdom), sectioned, mounted, stained, and visualized using a transmission electron microscope (H-7600; Hitachi, Tokyo, Japan). TEM was performed at the Hanaichi UltraStructure Research Institute (Okazaki, Aichi, Japan).

For the scanning transmission electron microscopy (STEM) tomography, the plastic-embedded sample was sectioned (700-nm thickness) and collected onto a Formvar-coated grid (Cu slot mesh). Colloidal gold particles (15 nm) were applied on both sides as a fiducial marker, and poststaining was performed with 2% uranyl acetate and 0.4% lead citrate. Single-axis STEM tomography was performed at an image size of 768 by 1,024 pixels using a JEM-2100F electron microscope (JEOL, Inc., Tokyo, Japan) operated at 200 kV. The tilt series were recorded at a pixel spacing of 4.56 nm on the specimen in a tilt range from −50° to +50° with an angular increment of 2°. The images obtained were aligned, and a three-dimensional (3-D) tomogram was generated using the IMOD software package (34). Segmentation in the 3-D tomogram was performed using Amira version 5.4.5 (Thermo Fisher Scientific, Waltham, MA).

### Cryo-EM.

A 2.5-μl virus suspension was applied onto an R 3.5/1 Cu Quantifoil grid (Quantifoil Micro Tools, Jena, Germany) that had been subjected to glow discharge beforehand. The grid was then blotted and plunge-frozen using a Vitrobot mark IV (Thermo Fisher Scientific) at 95% humidity and 4°C for 4 s. The frozen hydrated grid was mounted on a cryo-specimen holder (Gatan 626) at liquid nitrogen specimen temperature and imaged using a JEM2200FS electron microscope (JEOL, Inc.) equipped with a field-emission electron source operated at 200 kV. An in-column (omega-type) energy filter was used to enhance the image contrast in zero-energy-loss mode with a slit width of approximately 50 eV. The images were recorded on a DE20 direct detector (Direct Electron LP, USA) at a nominal magnification of ×20,000 in 15 movie frames with a total exposure time of 3 s. The total electron dose was less than 20 e^−^/Å^2^ for each image. The resultant pixel spacing was 2.82 Å. The recorded movie frames were subjected to motion correction using MotionCor2, following a previously described protocol (35).

### Scanning electron microscopy.

A. castellanii cells were cultured in PYG medium in 75-cm^2^ culture flasks and infected with cotonvirus or *Mimivirus shirakomae*. Four days after infection, virus particles in the culture medium were collected at 8,000 × *g* for 35 min at 4°C, resuspended in 5 ml of PBS, filtered through a 1.2-μm filter (GVS Filter Technology, Inc.), resuspended in 500 μl of 2% GA prepared in PBS, centrifuged, resuspended, and fixed with 50 μl of 2% GA in PBS. Finally, samples were visualized using a JSM-7500F microscope (JEOL, Japan) at the Hanaichi UltraStructure Research Institute (Okazaki, Aichi, Japan).

### Time-lapse imaging and kinetics analysis.

A. castellanii cells (5,000 per well) were seeded into a 24-well microplate, cultured in PYG medium, and exposed to viral particles (MOI = 100). Time-lapse images were captured using an all-in-one fluorescence microscope (BZ-X800/X810, Keyence Co.) with a 4× objective lens. In total, 2,160 frames were obtained, taken every minute for 36 h. Each frame was analyzed using a phase-contrast-based kinetic analysis algorithm for amoebae (PKA3) (30), which was developed in our laboratory.

### Immunofluorescence.

A. castellanii cells (1.0 × 10^5^) were cultured in PYG medium (2 ml) and exposed to viral particles (MOI = 100). After 8, 12, and 24 h, cells were collected by centrifugation at 500 × *g* for 5 min. The pellets were washed with PBS, fixed with 4% paraformaldehyde in PBS for 10 min at room temperature (RT; 22 ± 1°C), permeabilized using 0.1% Triton X-100 in PBS for 5 min, washed twice with 10 mM glycine in PBS for 5 min each time, blocked with 4% Block Ace (BA) for 30 min at room temperature (RT; 22°C), and incubated overnight at 4°C with anti-GM130 antibody [EP892Y] *cis*-Golgi marker (1:250 dilution in 0.4% BA solution) (ab52649; Abcam plc, Cambridge, UK). Cells were washed three times with 0.4% BA in 0.05% Tween 20, followed by incubation with goat anti-rabbit IgG H+L (1:500 dilution in 0.4% BA solution, Alexa Fluor 488; Abcam plc) for 1 h at RT and washing three more times. Finally, the cells were stained with 4′,6-diamidino-2-phenylindole (DAPI) (500 ng/ml) for 1 min at RT and washed with PBS for 5 min. Fluorescently labeled cells were observed using a BX50 microscope (Olympus, Tokyo, Japan) with a 100× oil objective lens. The images were merged using ImageJ (version 1.53a) (36).

### Genome sequencing and assembly.

Genomic DNA was prepared from viral particles using Nucleospin tissue XS (Macherey-Nagel GmbH and Co. KG, Germany) and sequenced on Illumina Novaseq 6000 and PacBio RS instruments, which was performed by Macrogen Japan (Koto-ku, Tokyo, Japan). Reads were assembled *de novo* using FALCON-integrate (version 2.1.4) (37), and a 1,476,464-bp genomic sequence was generated. Sequence reads were mapped to the genomic sequence using the CLC Genomics Workbench software (version 20.0.4) (Qiagen N.V., Hilden, Germany), and 17 regions with unusual sequence coverage were identified. These regions were confirmed using PCR and capillary sequencing. Finally, a 1,476,572-bp genomic sequence was obtained. The features of the viral genome were drawn using the CGView server (version 1.0) (38).

### Gene prediction and annotation.

Gene prediction was performed using Gene MarkS (39) and the FgenesV tool. tRNA genes were identified using the tRNAscan-SE server (version 2.0) (40). Amino acid homology searches were performed using BLASTp (NCBI non-redundant [nr] GenBank database) with an e-value threshold of <10^−5^, and gene annotation was manually revised. Annotated genes were manually classified into each category based on genome analysis using Nucleo-Cytoplasmic Virus Orthologous Group (NCVOG) proteins (41). 18S rRNA-like regions were predicted using BLASTn (NCBI 18S rRNA databases).

Homology searches of the 28-nucleotide-long Zamilon insert sequence (AATCTGATAATGAATCTGATAATGAATC), which has been observed previously in the mimivirus genome (15), were performed manually by searching the sequence on cotonvirus genome sequences in text-based searches. Conversely, nucleotide sequence homology searches between cotonvirus and MIMIVIRE-related sequences or virophages (Sputnik and Guarani) were performed using BLASTn (NCBI Nucleotide collection (nt) database).

### Phylogenetic analysis.

Nucleotide sequences of the family *Mimiviridae* were obtained from NCBI (Data Set S2) and aligned using the default option of the MUltiple Sequence Comparison by Log-Expectation (MUSCLE) program. All positions containing gaps and missing data were removed from the alignments, and the best evolutionary models were estimated. Phylogenetic trees were constructed using the maximum-likelihood method with the estimated best evolutionary models and 1,000 bootstrap replicates, and figures were drawn using the MEGA X program (version 10.0.5) (42). Concatenated alignments of NCLDV core genes, including those encoding the B family DNA polymerase, major capsid protein, D5-like ATPase, mRNA-capping enzyme, and virion-packaging ATPase, were manually joined after eliminating the gap. Then, the concatenated tree was reconstructed similarly to the individual trees.

The proteomic tree of NCLDVs was constructed using the ViPTree server (version 1.9) (29). This analysis was based on the Virus-Host DB and the manual addition of the following viral genomic nucleotide sequences: *Tupanvirus soda lake* (KY523104.2), *Tupanvirus deep ocean* (MF405918.2), *Bodo saltans virus* (MF782455.1), Acanthamoeba castellanii *medusavirus* (AP018495.1), faustovirus strain E12 (KJ614390.1), cedratvirus A11 (NC_032108.1), *Mollivirus sibericum* (NC_027867.1), and kaumoebavirus (NC_034249.1).

### Data availability.

The genome sequence of cotonvirus has been deposited in the DNA Data Bank of Japan (DDBJ) and GenBank (accession no. AP024483).

## References

[1] La Scola B, Audic S, Robert C, Jungang L, de Lamballerie X, Drancourt M, Birtles R, Claverie JM, Raoult D. 2003. A giant virus in amoebae. Science 299:2033. 10.1126/science.1081867. 12663918

[2] Raoult D, Audic S, Robert C, Abergel C, Renesto P, Ogata H, La Scola B, Suzan M, Claverie JM. 2004. The 1.2-megabase genome sequence of Mimivirus. Science 306:1344–1350. 10.1126/science.1101485. 15486256

[3] Claverie JM, Abergel C. 2018. Mimiviridae: an expanding family of highly diverse large dsDNA viruses infecting a wide phylogenetic range of aquatic eukaryotes. Viruses 10:506. 10.3390/v10090506. PMC6163669 30231528

[4] Arslan D, Legendre M, Seltzer V, Abergel C, Claverie JM. 2011. Distant Mimivirus relative with a larger genome highlights the fundamental features of Megaviridae. Proc Natl Acad Sci U S A 108:17486–17491. 10.1073/pnas.1110889108. 21987820 PMC3198346

[5] Yoosuf N, Yutin N, Colson P, Shabalina SA, Pagnier I, Robert C, Azza S, Klose T, Wong J, Rossmann MG, La Scola B, Raoult D, Koonin EV. 2012. Related giant viruses in distant locations and different habitats: *Acanthamoeba polyphaga* moumouvirus represents a third lineage of the Mimiviridae that is close to the Megavirus lineage. Genome Biol Evol 4:1324–1330. 10.1093/gbe/evs109. 23221609 PMC3542560

[6] Boyer M, Yutin N, Pagnier I, Barrassi L, Fournous G, Espinosa L, Robert C, Azza S, Sun S, Rossmann MG, Suzan-Monti M, La Scola B, Koonin EV, Raoult D. 2009. Giant Marseillevirus highlights the role of amoebae as a melting pot in emergence of chimeric microorganisms. Proc Natl Acad Sci U S A 106:21848–21853. 10.1073/pnas.0911354106. 20007369 PMC2799887

[7] Philippe N, Legendre M, Doutre G, Couté Y, Poirot O, Lescot M, Arslan D, Seltzer V, Bertaux L, Bruley C, Garin J, Claverie J-M, Abergel C. 2013. Pandoraviruses: amoeba viruses with genomes up to 2.5 Mb reaching that of parasitic eukaryotes. Science 341:281–286. 10.1126/science.1239181. 23869018

[8] Legendre M, Bartoli J, Shmakova L, Jeudy S, Labadie K, Adrait A, Lescot M, Poirot O, Bertaux L, Bruley C, Couté Y, Rivkina E, Abergel C, Claverie J-M. 2014. Thirty-thousand-year-old distant relative of giant icosahedral DNA viruses with a pandoravirus morphology. Proc Natl Acad Sci U S A 111:4274–4279. 10.1073/pnas.1320670111. 24591590 PMC3964051

[9] Legendre M, Lartigue A, Bertaux L, Jeudy S, Bartoli J, Lescot M, Alempic J-M, Ramus C, Bruley C, Labadie K, Shmakova L, Rivkina E, Couté Y, Abergel C, Claverie J-M. 2015. In-depth study of *Mollivirus sibericum*, a new 30,000-y-old giant virus infecting Acanthamoeba. Proc Natl Acad Sci U S A 112:E5327–E5335. 10.1073/pnas.1510795112. 26351664 PMC4586845

[10] Reteno DG, Benamar S, Khalil JB, Andreani J, Armstrong N, Klose T, Rossmann M, Colson P, Raoult D, La Scola B. 2015. Faustovirus, an Asfarvirus-related new lineage of giant viruses infecting amoebae. J Virol 89:6585–6594. 10.1128/JVI.00115-15. 25878099 PMC4468488

[11] Yoshikawa G, Blanc-Mathieu R, Song C, Kayama Y, Mochizuki T, Murata K, Ogata H, Takemura M. 2019. Medusavirus, a novel large DNA virus discovered from hot spring water. J Virol 93:e02130-18. 10.1128/JVI.02130-18. 30728258 PMC6450098

[12] Akashi M, Takemura M. 2019. Distribution of SNSs in Mimivirus genomes and the classification of mimiviruses isolated from Japan. Microbes Environ 34:451–455. 10.1264/jsme2.ME19077. 31645535 PMC6934397

[13] Schulz F, Alteio L, Goudeau D, Ryan EM, Feiqiao BY, Malmstrom RR, Blanchard J, Woyke T. 2018. Hidden diversity of soil giant viruses. Nat Commun 9:4881. 10.1038/s41467-018-07335-2. 30451857 PMC6243002

[14] Akashi M, Takemura M. 2019. Co-isolation and characterization of two pandoraviruses and a Mimivirus from a riverbank in Japan. Viruses 11:1123. 10.3390/v11121123. PMC6950457 31817274

[15] Levasseur A, Bekliz M, Chabrière E, Pontarotti P, La Scola B, Raoult D. 2016. MIMIVIRE is a defence system in Mimivirus that confers resistance to virophage. Nature 531:249–252. 10.1038/nature17146. 26934229

[16] Suzan-Monti M, La Scola B, Barrassi L, Espinosa L, Raoult D. 2007. Ultrastructural characterization of the giant volcano-like virus factory of Acanthamoeba Polyphaga Mimivirus. PLoS One 2:e328. 10.1371/journal.pone.0000328. 17389919 PMC1828621

[17] Mutsafi Y, Shimoni E, Shimon A, Minsky A. 2013. Membrane assembly during the infection cycle of the giant Mimivirus. PLoS Pathog 9:e1003367. 10.1371/journal.ppat.1003367. 23737745 PMC3667779

[18] Andrade AC, Rodrigues RA, Oliveira GP, Andrade KR, Bonjardim CA, La Scola B, Kroon EG, Abrahão JS. 2017. Filling knowledge gaps for Mimivirus entry, uncoating, and morphogenesis. J Virol 91:e01335-17. 10.1128/JVI.01335-17. PMC5660479 28878069

[19] Abrahão J, Silva L, Silva LS, Khalil JYB, Rodrigues R, Arantes T, Assis F, Boratto P, Andrade M, Kroon EG, Ribeiro B, Bergier I, Seligmann H, Ghigo E, Colson P, Levasseur A, Kroemer G, Raoult D, La Scola B. 2018. Tailed giant Tupanvirus possesses the most complete translational apparatus of the known virosphere. Nat Commun 9:749. 10.1038/s41467-018-03168-1. 29487281 PMC5829246

[20] Silva LC, Rodrigues RA, Oliveira GP, Dornas FP, La Scola B, Kroon EG, Abrahão JS. 2019. Microscopic analysis of the Tupanvirus cycle in Vermamoeba vermiformis. Front Microbiol 10:671. 10.3389/fmicb.2019.00671. 31001237 PMC6456662

[21] Francis R, Ominami Y, Khalil JY, La Scola B. 2019. High-throughput isolation of giant viruses using high-content screening. Commun Biol 2:216. 10.1038/s42003-019-0475-6. 31240254 PMC6584669

[22] Bowers B, Korn ED. 1969. The fine structure of Acanthamoeba castellanii (Neff strain). II. Encystment. J Cell Biol 41:786–805. 10.1083/jcb.41.3.786. 5768875 PMC2107820

[23] Silva LK, Rodrigues RA, Andrade AC, Hikida H, Andreani J, Levasseur A, La Scola B, Abrahão JS. 2020. Isolation and genomic characterization of a new Mimivirus of lineage B from a Brazilian river. Arch Virol 165:853–863. 10.1007/s00705-020-04542-5. 32052196

[24] Yutin N, Wolf YI, Koonin EV. 2014. Origin of giant viruses from smaller DNA viruses not from a fourth domain of cellular life. Virology 466–467:38–52. 10.1016/j.virol.2014.06.032. PMC4325995 25042053

[25] Koonin EV, Krupovic M, Yutin N. 2015. Evolution of double-stranded DNA viruses of eukaryotes: from bacteriophages to transposons to giant viruses. Ann N Y Acad Sci 1341:10–24. 10.1111/nyas.12728. 25727355 PMC4405056

[26] Schulz F, Yutin N, Ivanova NN, Ortega DR, Lee TK, Vierheilig J, Daims H, Horn M, Wagner M, Jensen GJ, Kyrpides NC, Koonin EV, Woyke T. 2017. Giant viruses with an expanded complement of translation system components. Science 356:82–85. 10.1126/science.aal4657. 28386012

[27] La Scola B, Desnues C, Pagnier I, Robert C, Barrassi L, Fournous G, Merchat M, Suzan-Monti M, Forterre P, Koonin E, Raoult D. 2008. The virophage as a unique parasite of the giant mimivirus. Nature 455:100–104. 10.1038/nature07218. 18690211

[28] Mougari S, Chelkha N, Sahmi-Bounsiar D, Pinto FD, Colson P, Abrahao J, La Scola B. 2020. A virophage cross-species infection through mutant selection represses giant virus propagation, promoting host cell survival. Commun Biol 3:248. 10.1038/s42003-020-0970-9. 32439847 PMC7242381

[29] Nishimura Y, Yoshida T, Kuronishi M, Uehara H, Ogata H, Goto S. 2017. ViPTree: the viral proteomic tree server. Bioinformatics 33:2379–2380. 10.1093/bioinformatics/btx157. 28379287

[30] Fukaya S, Aoki K, Kobayashi M, Takemura M. 2019. Kinetic analysis of the motility of giant virus-infected amoebae using phase-contrast microscopic images. Front Microbiol 10:3014. 10.3389/fmicb.2019.03014. 32038516 PMC6988830

[31] Nakamura N, Rabouille C, Watson R, Nilsson T, Hui N, Slusarewicz P, Kreis TE, Warren G. 1995. Characterization of a cis-Golgi matrix protein, GM130. J Cell Biol 131:1715–1726. 10.1083/jcb.131.6.1715. 8557739 PMC2120691

[32] Nakamura N, Lowe M, Levine TP, Rabouille C, Warren G. 1997. The vesicle docking protein p115 binds GM130, a cis-Golgi matrix protein, in a mitotically regulated manner. Cell 89:445–455. 10.1016/S0092-8674(00)80225-1. 9150144

[33] Takemura M. 2016. Morphological and taxonomic properties of Tokyovirus, the first Marseilleviridae member isolated from Japan. Microbes Environ 31:442–448. 10.1264/jsme2.ME16107. 27867160 PMC5158117

[34] Kremer JR, Mastronarde DN, McIntosh JR. 1996. Computer visualization of three-dimensional image data using IMOD. J Struct Biol 116:71–76. 10.1006/jsbi.1996.0013. 8742726

[35] Zheng SQ, Palovcak E, Armache JP, Verba KA, Cheng Y, Agard DA. 2017. MotionCor2: anisotropic correction of beam-induced motion for improved cryo-electron microscopy. Nat Methods 14:331–332. 10.1038/nmeth.4193. 28250466 PMC5494038

[36] Schneider CA, Rasband WS, Eliceiri KW. 2012. NIH Image to ImageJ: 25 years of image analysis. Nat Methods 9:671–675. 10.1038/nmeth.2089. 22930834 PMC5554542

[37] Chin C-S, Peluso P, Sedlazeck FJ, Nattestad M, Concepcion GT, Clum A, Dunn C, O’Malley R, Figueroa-Balderas R, Morales-Cruz A, Cramer GR, Delledonne M, Luo C, Ecker JR, Cantu D, Rank DR, Schatz MC. 2016. Phased diploid genome assembly with single-molecule real-time sequencing. Nat Methods 13:1050–1054. 10.1038/nmeth.4035. 27749838 PMC5503144

[38] Stothard P, Wishart DS. 2005. Circular genome visualization and exploration using CGView. Bioinformatics 21:537–539. 10.1093/bioinformatics/bti054. 15479716

[39] Besemer J, Lomsadze A, Borodovsky M. 2001. GeneMarkS: a self-training method for prediction of gene starts in microbial genomes. Implications for finding sequence motifs in regulatory regions. Nucleic Acids Res 29:2607–2618. 10.1093/nar/29.12.2607. 11410670 PMC55746

[40] Lowe TM, Chan PP. 2016. TRNAscan-SE On-line: integrating search and context for analysis of transfer RNA genes. Nucleic Acids Res 44:W54–W57. 10.1093/nar/gkw413. 27174935 PMC4987944

[41] Yutin N, Wolf YI, Raoult D, Koonin EV. 2009. Eukaryotic large nucleo-cytoplasmic DNA viruses: clusters of orthologous genes and reconstruction of viral genome evolution. Virol J 6:223. 10.1186/1743-422X-6-223. 20017929 PMC2806869

[42] Kumar S, Stecher G, Li M, Knyaz C, Tamura K. 2018. MEGA X: Molecular evolutionary genetics analysis across computing platforms. Mol Biol Evol 35:1547–1549. 10.1093/molbev/msy096. 29722887 PMC5967553

