# Supplementary material for: Morphological and Taxonomic Properties of the Newly Isolated Cotonvirus japonicus, a New Lineage of the Subfamily Megavirinae
Source: J Virol. 2021 Aug 25;95(18):e00919-21. doi: 10.1128/JVI.00919-21 (PMC8387033; doi:10.1128/JVI.00919-21)
Supplement: Supplemental file 1 — Supplemental Data Set and Movie Legends. Download JVI.00919-21-s0005.pdf, PDF file, 0.1 MB [file jvi.00919-21-s0005.pdf]

Supplemental Materials

Title: Morphological and taxonomic properties of the newly isolated *Cotonvirus japonicus*, a new lineage of the subfamily *Megavirinae*

Authors: Haruna Takahashi, Sho Fukaya, Chihong Song, Kazuyoshi Murata, and Masaharu Takemura

Corresponding author: Masaharu Takemura (giantvirus@rs.tus.ac.jp)

Supplemental data 1

Supplemental data 2

Supplemental movie 1

Supplemental movie 2

Supplemental data 1

Predicted ORFs of *Cotonvirus japonicus*.

Supplemental data 2

Accession no. of genes, which were used in molecular phylogenetic analyses.

Supplemental movie 1

Time lapse movies used in the kinetic analysis of *A. castellanii* cells infected with mimivirus (*Mimivirus shirakomae*), megavirus (*Megavirus musashi*), and cotonvirus, respectively. In total, 2,160 frames were obtained, each taken every minute for 36 h. The 1 s of this movie is 1 h of real time.

Supplemental movie 2

Tomogram slices and segmentations of the VFs in a cotonvirus-infected amoeba within a 700 nm-thick section. light blue: VFs, Blue: tubular-structures, Orange: the center core of VF.
